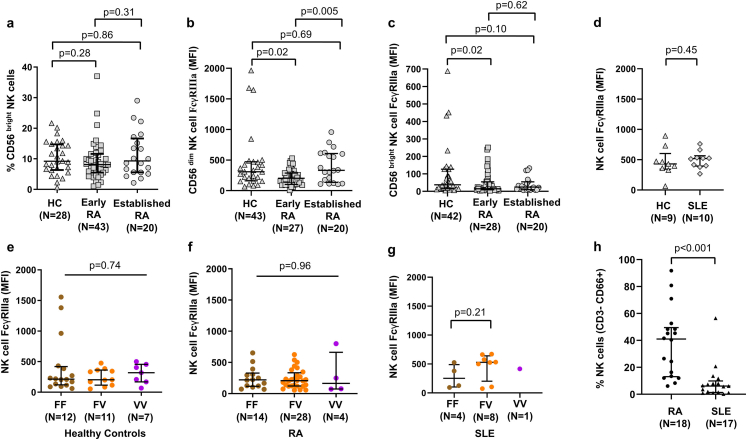# Comprehensive genetic and functional analyses of Fc gamma receptors influence on response to rituximab therapy for autoimmunity

**DOI:** 10.1016/j.ebiom.2022.104343

**Published:** 2022-11-11

**Authors:** James I. Robinson, Md Yuzaiful Md Yusof, Vinny Davies, Dawn Wild, Michael Morgan, John C. Taylor, Yasser El-Sherbiny, David L. Morris, Lu Liu, Andy C. Rawstron, Maya H. Buch, Darren Plant, Heather J. Cordell, John D. Isaacs, Ian N. Bruce, Paul Emery, Anne Barton, Timothy J. Vyse, Jennifer H. Barrett, Edward M. Vital, Ann W. Morgan, MATURA Consortia, MASTERPLANS Consortia

**Affiliations:** a School of Medicine, University of Leeds and NIHR Leeds Biomedical Research Centre, Leeds Teaching Hospitals NHS Trust, UK; b School of Mathematics and Statistics, University of Glasgow, UK; c Cancer Research UK Cambridge Institute, University of Cambridge, UK; d Department of Biosciences, School of Science and Technology, Nottingham Trent University, UK; e Department of Clinical Pathology, Faculty of Medicine, Mansoura University, Mansoura, Egypt; f Department of Medical and Molecular Genetics, Faculty of Life Sciences and Medicine, King's College London, UK; g Haematological Malignancy Diagnostic Service, Leeds Teaching Hospitals NHS Trust, UK; h Versus Arthritis Centre for Genetics and Genomics, Division of Musculoskeletal and Dermatological Sciences, School of Biological Sciences, The University of Manchester and NIHR Manchester BRC, Manchester University NHS Foundation Trust, UK; i Population Health Sciences Institute, Newcastle University, UK; j Translational and Clinical Research Institute, Newcastle University and Musculoskeletal Unit, Newcastle upon Tyne Hospitals NHS Foundation Trust, UK; k NIHR Leeds Medtech and In vitro Diagnostics Co-operative, Leeds Teaching Hospitals NHS Trust, UK

**Keywords:** Autoimmune diseases, B-lymphocytes, Genetics, Rheumatoid arthritis, Rituximab, Systemic lupus erythematosus

## Abstract

**Background:**

Rituximab is widely used to treat autoimmunity but clinical response varies. Efficacy is determined by the efficiency of B-cell depletion, which may depend on various Fc gamma receptor (FcγR)-dependent mechanisms. Study of FcγR is challenging due to the complexity of the *FCGR* genetic locus. We sought to assess the effect of *FCGR* variants on clinical response, B-cell depletion and NK-cell-mediated killing in rheumatoid arthritis (RA) and systemic lupus erythematosus (SLE).

**Methods:**

A longitudinal cohort study was conducted in 835 patients [RA = 573; SLE = 262]. Clinical outcome measures were two-component disease activity score in 28-joints (2C-DAS28CRP) for RA and British Isles Lupus Assessment Group (BILAG)-2004 major clinical response (MCR) for SLE at 6 months. B-cells were evaluated by highly-sensitive flow cytometry. Single nucleotide polymorphism and copy number variation for genes encoding five FcγRs were measured using multiplex ligation-dependent probe amplification. *Ex vivo* studies assessed NK-cell antibody-dependent cellular cytotoxicity (ADCC) and FcγR expression.

**Findings:**

In RA, carriage of *FCGR3A*-158V and increased *FCGR3A*-158V copies were associated with greater 2C-DAS28CRP response (adjusted for baseline 2C-DAS28CRP). In SLE, MCR was associated with increased *FCGR3A*-158V, OR 1.64 (95% CI 1.12–2.41) and *FCGR2C*-ORF OR 1.93 (95% CI 1.09–3.40) copies. 236/413 (57%) patients with B-cell data achieved complete depletion. Homozygosity for *FCGR3A*-158V and increased *FCGR3A*-158V copies were associated with complete depletion in combined analyses. *FCGR3A* genotype was associated with rituximab-induced ADCC, and increased NK-cell FcγRIIIa expression was associated with improved clinical response and depletion *in vivo*. Furthermore, disease status and concomitant therapies impacted both NK-cell FcγRIIIa expression and ADCC.

**Interpretation:**

FcγRIIIa is the major low affinity FcγR associated with rituximab response. Increased copies of the *FCGR3A*-158V allele (higher affinity for IgG1), influences clinical and biological responses to rituximab in autoimmunity. Enhancing FcγR-effector functions could improve the next generation of CD20-depleting therapies and genotyping may stratify patients for optimal treatment protocols.

**Funding:**

10.13039/501100000265Medical Research Council, National Institute for Health and Care Research, Versus Arthritis.

**Research in context**

**Evidence before this study**

Mechanistic studies to explain the variability in depth of B-cell depletion by rituximab therapy in autoimmune diseases, including the effect of *FCGR* variants, were limited compared to the literature in haematological malignancies. Study of FcγRs is challenging due to the complexity of the *FCGR* locus and large samples of rituximab-treated individuals with highly-sensitive flow cytometry B-cell data are required. We searched PubMed, Cochrane Library and medRxiv for articles published in English up to March 2022 using the following terms: “fc receptor”, “rituximab”, “rheumatoid arthritis”, and “systemic lupus erythematosus” and identified 35 studies. Of those studies, only 7 studies (RA = 6; SLE = 1) evaluated the effect of *FCGR* genotype on clinical outcomes. One meta-analysis which only included 3 studies in RA reported an association between the *FCGR3A* VV+VF genotype and rituximab response. However, the genetic studies in autoimmune diseases above have in general, lacked statistical power (sample sizes between 12 and 212), included heterogeneous cohorts, treatment regimens, and outcome measures, as well as genotyping technologies that were neither comprehensive nor accounted for copy number variation (CNV).

**Added value of this study**

By undertaking comprehensive assessment of all low affinity *FCGR* variants and CNV in the largest RA and SLE cohorts to date, we showed that FcγRIIIa is the major FcγR contributing to rituximab biological (i.e. depth of B-cell depletion) and clinical responses in both autoimmune diseases. Our genetic findings were supported by *ex vivo* data characterising FcγRIIIa expression and NK-cell-mediated cytotoxicity. In SLE, our study shows the association between increased copies of the *FCGR2C*-ORF allele and improved clinical response.

**Implications of all the available evidence**

Our findings indicate that personalised therapy could be guided by *FCGR3A* genotyping for optimal treatment protocols which may reduce complications such as neutropaenia, and infections related to hypogammaglobulinaemia that develops with repeated courses of rituximab therapy. Enhancing the FcγR-effector functions could improve the next generation of CD20-depleting therapies with a focus on enhancing ADCC for *FCGR3A*-158F homozygotes.

## Introduction

B-cell depletion using rituximab is widely used to treat rheumatoid arthritis (RA) and systemic lupus erythematosus (SLE).^1^, ^2^, ^3^, ^4^ However, clinical responses vary and there remains an unmet need to understand mechanisms of sub-optimal response to improve clinical outcomes. Significant evidence supports an association between achieving complete peripheral B-cell depletion and clinical response in RA and SLE, when highly sensitive flow cytometry (HSFC) assays are used to enumerate circulating B-cells.^5^, ^6^, ^7^, ^8^, ^9^ However, mechanistic studies to explain this variability in depth of depletion in autoimmunity are limited.

Rituximab is a chimeric anti-CD20 monoclonal antibody (mAb), with a native IgG1-Fc that crosslinks Fcγ receptors (FcγRs) expressed on immune effector cells. Variability in B-cell depletion traditionally invokes four main mechanisms, and the relative importance of each may differ between autoimmunity and malignancies. These include antibody-dependent cellular cytotoxicity (ADCC), complement-dependent cytotoxicity (CDC), antibody-dependent phagocytosis (ADCP) and direct signalling-induced cell death, with variable evidence from animal models, *in vitro* and clinical studies.^10^^,^^11^ Human genetic studies provide strong evidence for natural killer (NK)cell-mediated ADCC, delivered through FcγRIIIa, as the principal mechanism.^10^^,^^12^^,^^13^

The expression of FcγR subtypes is known to differ between leucocyte populations. NK-cells are generally characterised by expression of FcγRIIIa, with greater expression in the circulation than in tissues. NK-cells from some individuals express FcγRIIc, or rarely FcγRIIb dependent upon specific gene rearrangements.^14^ In tissues, other FcγR-mediated mechanisms and innate cells may also contribute, such as ADCP, which leads to clearance of rituximab-opsonised B-cells by cells of the reticuloendothelial system, tissue macrophages^15^ or neutrophils.^12^^,^^16^ Phagocytic cells express activatory FcγRs (i.e. FcγRIIa, FcγRIIc, FcγRIIIa and FcγRIIIb) in a cell-type specific manner. Innate cell activation may also lead to release of soluble mediators that modulate FcγR expression on phagocytic cells, for example interferon gamma (IFNγ) release from activated NK-cells and complement component 5a (C5a) release from Kupffer cells exposed to IgG-coated B-cells, potentially further enhancing FcγR-mediated clearance mechanisms.^11^^,^^13^^,^^17^ The importance of macrophage-mediated ADCP is well-recognised in haematological malignancies, with promising early results for a combination of a macrophage checkpoint inhibitor and rituximab in follicular lymphoma.^18^ Furthermore, a recent study of cancer immunotherapy demonstrated the importance of macrophage polarisation and identified that paclitaxel acted as an adjuvant to polarise macrophages to the M1 phenotype with enhanced phagocytic capacity,^16^ including ADCP. There is a single inhibitory FcγR (FcγRIIb), which fine-tunes activatory signals. FcγRIIb is the only FcγR expressed on B-cells where it may contribute to rituximab-mediated CD20 internalisation.^19^ Differences in the relative importance of FcγR-effector functions between different disease states and tissue sites may ultimately be explained by inherent differences in IgG and immune complex structure, B-cell biology, abundance of tissue/tumour associated macrophages, immune cell polarisation, concomitant immunosuppressive medications and/or adjuvants.

Evolutionary gene duplications and rearrangements created a structurally variable *FCGR* genetic locus, with duplications and deletions observed.^14^^,^^20^^,^^21^ Due to the high homology between paralogs, *FCGR* genotyping is technically challenging. There are well-described functional variants that alter FcγR-IgG affinity and/or FcγR expression, which may modulate IgG-effector functions leading to rituximab-induced B-cell depletion and clinical response. Some studies have evaluated genetic predictors of rituximab response in genes encoding the low affinity FcγRs in RA,^22^, ^23^, ^24^, ^25^, ^26^ SLE^27^ and rituximab-induced neutropenia.^28^^,^^29^ However, these studies have, in general, lacked statistical power, included heterogeneous cohorts, treatment regimens, outcome measures and genotyping strategies, which confounds meta-analyses.^30^ Despite associations with malignancies,^31^, ^32^, ^33^, ^34^, ^35^ these were not replicated in larger cohorts from the RESORT,^36^ PRIMA,^37^ GOYA, or GALLIUM^38^ clinical trials. Candidate variants included *FCGR3A* (F158V, rs3969910) and *FCGR2A* (H131R, rs1801274), which encode receptors with single amino acid differences in the IgG binding sites. The FcγRIIIa-158V and FcγRIIa-131H allotypes have increased affinity for IgG1 and IgG2, respectively. We and others have shown that *FCGR3A*^39^ and *FCGR3B* CNV^40^^,^^41^ correlate with cell surface expression, which may further modulate B-cell depletion. To date, there have been no studies of rituximab response that have comprehensively studied common *FCGR* variants nor accounted for copy number variation (CNV).

To provide a mechanistic explanation for the clinical importance of rituximab-FcγR engagement, we aimed to assess association of FcγR allotypes on B-cell depletion, clinical response and *ex vivo* NK-cell-mediated killing with a view to informing personalised B-cell depleting therapies in autoimmunity. Our genotyping approach provided the opportunity to take account of CNV and explore both gene and allele copy number (CN).

## Methods

In order to make replication of our work simpler, we have adopted a commercial multiplexed ligation-dependent probe amplification (MLPA) platform^39^ for measuring genetic variation at the *FCGR* locus, supplemented with our in-house *FCGR2C* quantitative sequence variant (QSV) assay, which together offer combined measures of qualitative single nucleotide polymorphisms (SNPs) and quantitative CNVs.

### Study design

Prospective and retrospective longitudinal cohort studies were conducted in patients with RA and SLE who were treated with rituximab from January 2001 to January 2020. These patients were recruited from two UK biologic DMARD (bDMARD) registries and a large cohort from Leeds Teaching Hospitals NHS Trust (LTHT).

### Study population: rheumatoid arthritis

231 patients with RA were recruited from the Biologics in RA Genetics and Genomics Study Syndicate (BRAGGSS), which collected blood samples from bDMARD-treated RA patients recruited into British Society for Rheumatology Biologics Register for RA (www.braggss.co.uk). A further 342 RA patients were recruited from the LTHT biologics clinic. Inclusion criteria were a consultant diagnosis of RA; adults (≥18 years) at symptom onset of RA; and fulfilling the minimal clinical dataset criteria to be included in the downstream efficacy analyses (availability of 2C-DAS28CRP data at baseline and 3–6 months post-rituximab in Cycle 1 of therapy OR availability of peripheral B-cells data at baseline and post-rituximab in Cycle 1 of therapy AND have DNA of suitable quality for genotyping).

For functional studies, peripheral blood were obtained from three separate cohorts from LTHT; (i) DMARD-experienced adult RA patients with long-standing disease (>2 years), (ii) DMARD-naïve early RA patients (i.e. symptom duration <1 year) who were recruited into the Infliximab as Induction Therapy in Early RA (IDEA) study, as previously published^42^; and (iii) rituximab-treated RA patients from biologics clinic, where blood was taken immediately prior to first rituximab cycle.

### Study population: systemic lupus erythematosus

177 patients with SLE were recruited from the British Isles Lupus Assessment Group (BILAG) Biologics Registry (BILAG-BR). While the other 85 patients were recruited from the connective tissue disease clinic at LTHT. Inclusion criteria were adults (≥18 years); fulfilling the revised 1997 American College of Rheumatology classification for SLE; active SLE as defined by 1 x BILAG A or 2 x BILAG B grades; and fulfilling the minimal clinical dataset criteria to be included in the downstream efficacy analyses (availability of BILAG response data at baseline and 4–6 months post-rituximab in Cycle 1 of therapy OR availability of peripheral B-cells data at baseline and post-rituximab in Cycle 1 of therapy AND have DNA of suitable quality for genotyping). For the functional studies, peripheral blood was obtained immediately prior to first cycle rituximab in some SLE patients.

### Ethics statement

All studies were approved by a Research Ethics Committee (REC); North West Multicentre (00/8/053), North West Greater Manchester (09/H1014/64), COREC (04/Q1403/37), Leeds West (01/023, 05/MRE03/85 and 09/H1307/98) and Leeds East (04/Q1206/107, 10/H1306/88). All participants provided informed written consent.

### Treatment

All patients received a first cycle of therapy consisting of 100 mg of methylprednisolone and 1000 mg of rituximab given intravenously on days 1 and 14, with the exception of 3 RA patients who received 1g of rituximab in total. All RA patients received rituximab MabThera® while 7/262 SLE patients received a rituximab biosimilar. Continuation of a stable dose or reduction of concomitant DMARDs and/or oral prednisolone was left to the clinicians’ discretion with the aim to stop glucocorticoids if remission was achieved within 6 months.

### Clinical outcomes: RA

We used a validated two-component disease activity score in 28-joints (2C-DAS28CRP), adjusted to baseline 2C-DAS28CRP as our primary outcome measure. Previous pharmacogenetic studies showed that inclusion of subjective measures (tender joint count and visual analogue scale of global health assessment in the DAS28 score) impeded the identification of relevant genetic markers of TNF-inhibitor (TNFi) response.^43^^,^^44^ Clinical outcome measurements (swollen joint count based on 28-joint assessment (SJC28) and CRP, in mg/L) were taken at baseline and ∼6-months post-rituximab. Where CRP levels were recorded as <5 mg/L (lower limit of reliable detection) these were replaced by imputed values from a Uniform distribution from 0 to 5. The 2C-DAS28CRP scores were calculated using the formula: [√SJC28 + (0.6 x ln (CRP + 1))].^45^ The three-component disease activity score based on 28 joints and CRP (3C-DAS28CRP) was calculated for comparative purposes using the formula: 1.10 x [(0.56 x √TJC28) + (0.28 x SJC28) + (0.36 ln(CRP+1))].^46^

### Clinical outcomes: SLE

Disease activity was assessed using the BILAG-2004 index at baseline and approximately every 6 months thereafter. Clinical responses at 6-months were standardised between the two cohorts and determined as follows: (i) MCR = improvement of all domains rated A/B to grade C/better and no A/B flare between baseline and 6-months;^2^ PCR = maximum of 1 domain with a persistent grade B with improvement in all other domains and no A or B flare; and^3^ non-response = those not meeting the criteria for either MCR or PCR(8).

### Routine laboratory assessments

All autoantibody and immunoglobulin assessments were determined using standard assays in the routine NHS diagnostics laboratory of each participating site. RA patients who ever had RF and ACPA titres of ≥40 iu/mL and ≥7 iu/mL, respectively, were defined as positive, to maintain consistency with previously published studies. For SLE, ANA was tested using indirect immunofluorescence and a panel of nuclear autoantibodies including anti-dsDNA and anti-ENA antibodies (Ro52, Ro60, La, Sm, and RNP). Complement (C3 and C4) and total serum immunoglobulin (IgM, IgA and IgG) titres were measured by nephelometry. Adult reference ranges are as follows: C3: 0.75–1.65 g/L, C4: 0.14–0.54 g/L), IgG (6–16 g/L), IgA (0.8–4 g/L) and IgM (0.5–2 g/L).

### Highly-sensitive flow cytometry

Peripheral blood B-cell subsets were measured using HSFC at the accredited Leeds Haematological Malignancy Diagnostic Service at baseline and on follow-up, as previously described.^5^ A six-colour flow cytometry protocol (CD3, CD14, CD19, CD27, CD38, CD45), counting 500,000 events was used. Naive (CD19+CD27-), memory (CD19++CD27+) and plasmablast (CD19+/-CD27++CD38++) counts were enumerated using CD45 to identify the total leucocyte population for calculation of absolute B-cell subset numbers, using CD3 and CD14 to exclude contaminating leucocyte populations. Complete B-cell depletion was defined as a total B-cell count ≤0.0001 × 10^9^ cells/L at week 2 for RA and week 6 for SLE.

### Genotyping

Genomic DNA was extracted from EDTA-anticoagulated whole blood using Qiamp mini spin columns (Qiagen), Gene Catcher (ChargeSwitch, Thermo Fisher) and a manual phenol chloroform method. DNA concentration was measured using UV spectrophotometry and samples diluted to 10 ng/μl. 50 ng total template was used for each MLPA reaction. MLPA probe mix panels P110 and P111 version B2 (MRC-Holland, Amsterdam, The Netherlands) were performed for every sample.

Where a sample failed in the first reaction due to insufficient template (Q fragment QC) the DNA template was concentrated using ethanol precipitation and MLPA repeated. Amplified MLPA fragments were separated against a LIZ GS 5500 size standard using an ABI 3130 Applied Biosystems (Warrington, UK), instrument fitted with a 36 cm 16 capillary array. Intra-sample normalisation against internal amplification controls and reference probes was performed using Coffalyser.NET software (MRC-Holland), prior to inter sample normalisation in batch analysis mode. Interpretation of the underlying gene copy number and qualitative variants followed the guidelines set out in the product information sheets, with the exception of *FCGR2C* as described below.

For RA, 573 patients were genotyped. A sample size of N = 481 would provide >80% power to detect a variant explaining 2.5% of outcome variance at a nominal significance level of 0.01. A sample size of >1200 patients would provide >80% power to detect a variant explaining ∼1% variance.

### *FCGR2C* copy number assay

High sequence identity between *FCGR2A*, *FCGR2B* and *FCGR2C* prevented the MLPA probes in panels P110 and P111 from uniquely recognising the copy number variable *FCGR2C* without simultaneously hybridising to *FCGR2B* or *FCGR2A*. Interpretation of *FCGR2C* gene copy number required multiple probe intensities to be combined. In our hands, these MLPA probes lacked the consistency required to confidently call *FCGR2C* copy number.

Using a gene-specific resequencing approach in 32 individual genomes we surveyed all *FCGR2* variants and classified them as either paralogous sequence variants (PSVs) or SNPs. Long-range PCR was used to amplify each gene specifically using the oligonucleotide primers in Supplementary Table S1. We identified a single PSV that differentiated all three genes (Supplementary Fig. S1a). To supplement the MLPA panels we developed a QSV assay to measure the copy number of *FCGR2C* directly referenced to both *FCGR2A* and *FCGR2B*. A single pair of primers was used to co-amplify 279bp fragments of all three genes with equal efficiency in a single reaction. PCR cycles (annealing at 55 °C, 1.5 mM MgCl2) were limited to 30, to maintain proportional amplification of all three genes. Amplicons were purified and concentrated and normalised using a modified ChargeSwitch (Thermo Fisher, Warrington, UK) process, utilising a limiting quantity of magnetic beads and eluting in a fifth of the original volume. Purified amplicons were Sanger sequenced using the reverse primer, and relative electropherogram peak heights of gene specific PSVs were determined using QSVanalyser software.^47^ Comparison of the 2C-derived peak height with the invariant 2B and 2A, allowed copy number estimation with respect to two reference genes (Supplementary Fig. S1b). Copy number of *FCGR2C* was inferred from cluster centres as indicated.

**Fig. 1:**
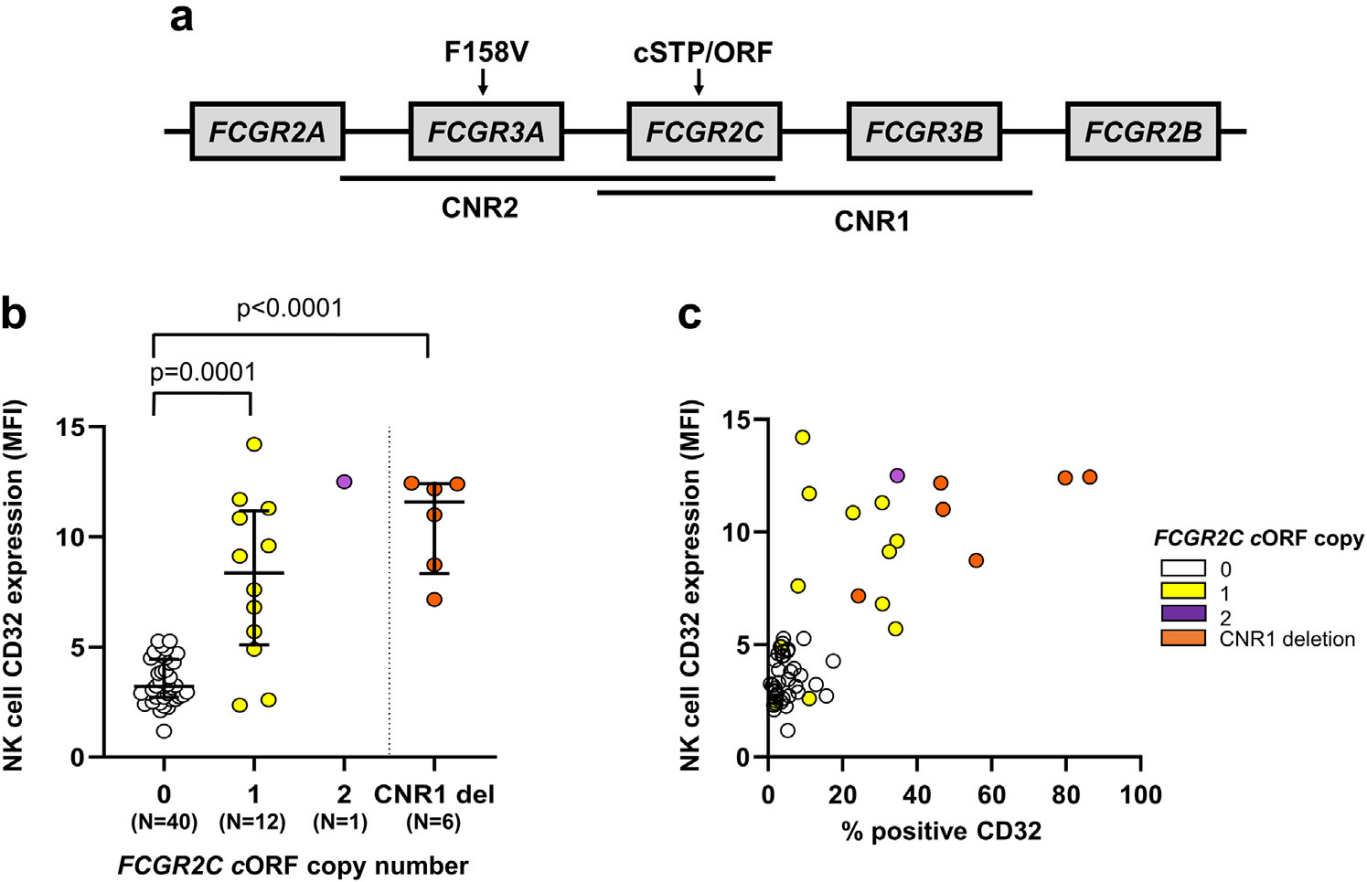
***FCGR2C* QSV assay is associated with CD32 expression on NK-cells.(a)** Schematic of the two main copy number regions (CNR1 and 2) and the relative positions of the functionally relevant nonsynonymous variants affecting *FCGR3A* and *FCGR2C*. **(b)** Expression of CD32 on NK-cells in treatment-naïve, early RA patients (n = 59) stratified by *FCGR2C* open reading frame (ORF) copy number. Data were summarised as median and the error bars denoted interquartile range. Using our *FCGR2C* QSV assay (Supplementary Fig. S1), in combination with multiplex ligation dependent assay probe values for rs10917661, copies of *FCGR2C*-ORF were genotyped where matched NK-cell CD32 (clone KB61) expression data were available. It was not possible to distinguish classical and non-classical *FCGR2C*-ORF in our study. The latter variant also contains a premature STP codon, precluding FcγRIIc translation. A particular locus rearrangement including a deletion of one copy of *FCGR2C* and one copy of *FCGR3B* (CNR1 del), has been described to lead to expression of FcγRIIb on NK-cells. The rearrangement was observed in conjunction with *FCGR2C*-STP in 5/6 individuals, where we would anticipate FcγRIIc would be the only class II FcγR expressed. **(c)** The relationship between % positive CD32 expression on NK-cells, and geometric mean fluorescence intensity (MFI) in treatment-naïve, early RA patients (n = 59). P-values calculated using non-parametric Mann–Whitney test. Data are summarised using median and interquartile range.

The genotype, Hardy Weinberg equilibrium, gene copy number and copy number region (CNR) loss and gain frequencies, which summarise *FCGR* locus rearrangements (Fig. 1a), for each of our cohorts are shown in Supplementary Tables S2, S3, and S4, respectively. Our data are consistent with the published literature^48^ including measures of linkage equilibrium, which are presented from diploid British Caucasian SLE individuals (Supplementary Table S5).

### Flow cytometry and NK-cells

NH_4_Cl lysed heparinised blood samples for RA and PBMCs for SLE were incubated with 10 μg/ml human IgG (Novartis, Hanover, Germany), to block non-specific binding, for 30 min on ice. Cells were stained (30 min, on ice, in dark) with anti-CD16-PE (clone 3G8, Caltag-Medsystems, Buckingham, UK), anti-CD32 (clone KB61, Dako, Cambridge UK), anti-CD3-TR (S4.1, Caltag-Medsystems, Buckingham, UK) and anti-CD56-PerCP (B159, Becton Dickinson Biosciences, Berkshire, UK) or isotype controls for experiments in RA and matched HC, while cells were stained with anti-CD16-APC Vio770 (REA423, Miltenyi Biotec, Surrey, UK), anti-CD3-VioGreen (REA613, Miltenyi Biotec, Surrey, UK) and anti-CD56-VioBright FITC (AF12-7H3, Miltenyi Biotec, Surrey, UK) or isotype controls for experiments in SLE and matched HC. Cells were then washed in ice cold FACS Buffer and 5000 events were acquired within the appropriate forward scatter (FSC)/side scatter (SSC) and CD3-gates on FACSCalibur (BD Biosciences, Berkshire, UK). Peripheral blood NK-cells (CD3-CD56+) were identified on the basis of lymphocyte FSC/SSC and specific gating for lack of expression of CD3 and positive expression of CD56 (all NK-cells, Supplementary Fig. S2a). Expression of FcγRIIIa on NK-cells (CD3-CD56+CD16+) were evaluated by geometric mean fluorescence intensity (MFI). The CD16/CD32 geometric MFI and the percentage of CD16/CD32 positive cells were determined. NK-cell subsets were distinguished by separate gates created around CD56^dim^/CD16++ and CD56^bright^/CD16^neg/low^ NK-cells (Supplementary Fig. S2b). Absolute NK numbers (cells/μL) = Lymphocyte count (cell number/μL of the blood count) x proportion of the cell subpopulation of interest ÷ 100. Adult reference ranges are 90–600 cells/μL.^49^

### NK-cell degranulation assays

#### Rheumatoid arthritis and matched controls

Pre-genotyped, thawed cryopreserved PBMCs isolated from NH_4_Cl-lysed heparinised blood using a Ficoll–Paque gradient were recovered overnight in RPMI Glutamax media (Invitrogen, Loughborough, UK) containing 10% FBS at a concentration of 2 × 10^6^ cells/ml in the presence of 400U/ml of IL-2 (Sigma, Dorset, UK). All experiments were performed at 37 °C in a humidified 5% CO_2_ incubator, unless otherwise stated. NK-cell degranulation assays were performed over 4 h in the presence of CD107a FITC antibody (H4A3; BD Biosciences, Berkshire, UK) using recovered PBMCs and human B lineage cell lines (Daudi cells; ATCC, Manassas, Virginia, USA) that had been pre-incubated with rituximab (Roche Ltd, Basel, Switzerland) at 10 μg/ml overnight using effector-target (E:T) ratios of 1:1. GolgiStop™ (BD Biosciences, Berkshire, UK) was added after 1 h to halt protein transport and prevent internalisation of CD107a. Negative controls included Daudi cells incubated overnight in the absence of rituximab (E:T of 1:1; spontaneous degranulation) and assays performed without CD107a in the presence of a FITC-isotype control (Dako, Santa Clara, USA). After 4 h, cells were washed and stained with anti-CD3 (S4.1; Invitrogen, Loughborough, UK), anti-CD14 (MφP9; BD Biosciences, Berkshire, UK) and anti-CD56 (B159; BD Biosciences, Berkshire, UK) for 30 mins (on ice, in dark) then washed in ice cold FACS buffer and flow cytometric analysis was performed as outlined above. The ratio of CD107a positive NK-cells without:with rituximab, identified by FSC/SSC, CD3 and CD56 expression, was calculated using the Flowjo software analysis package (BD Biosciences, Berkshire, UK). Additional blocking assays, were performed in a subset of experiments with anti-CD16 (3G8, Invitrogen, Loughborough, UK), anti-CD32 (KB61, Dako, Santa Clara, USA) and mouse IgG (Invitrogen, Loughborough, UK).

#### Systemic lupus erythematosus and matched controls

For experiments performed on SLE cases and matched controls, fresh PBMCs were initially isolated using Greiner Bio-One Leucosep™ centrifuge tubes with porous barrier (Thermo Fisher Scientific, Loughborough, UK). NK-cells were isolated by negative selection using an NK-cell Isolation Kit and Immunomagnetic (Miltenyi Biotec, Surrey, UK) and cytofluorometric selection of CD3-CD56+ NK-cells using flow cytometry resulting in ≥90% purity. During optimisation, autologous B-cells were also isolated from PBMCs of 7 SLE patients and 5 healthy controls by positive selection using a B-cell Isolation Kit II and Immunomagnetic (Miltenyi Biotec, Surrey, UK).

**Fig. S3:**
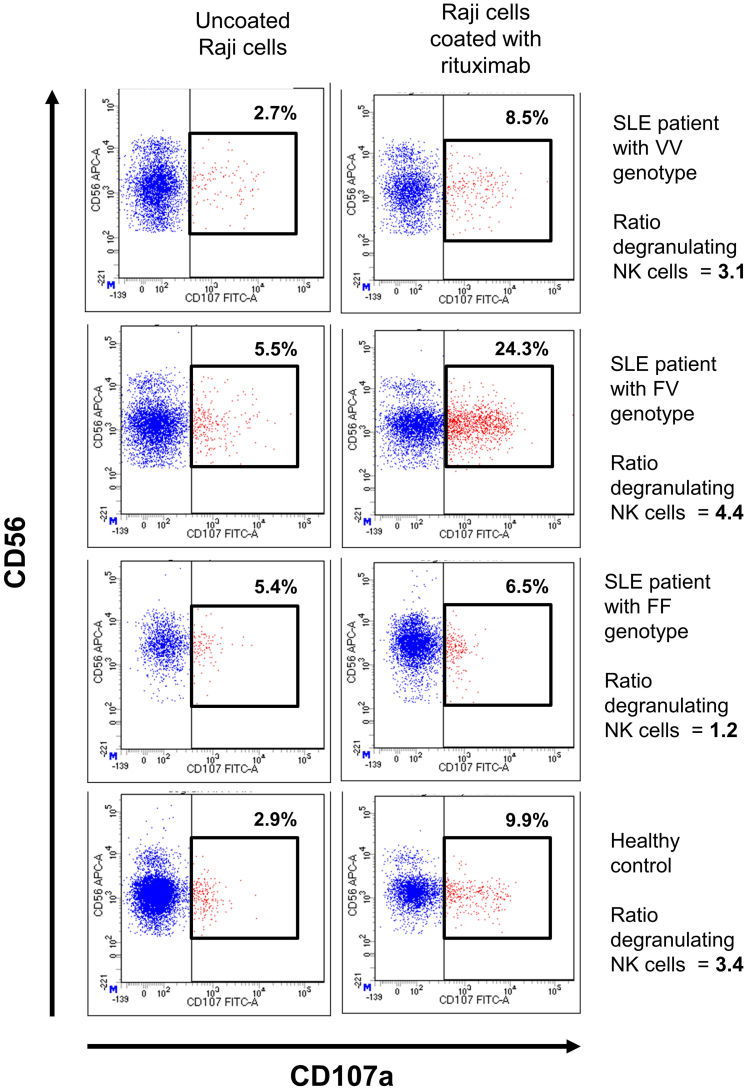


NK-cell degranulation assays were performed by the addition of Rituximab (N7049B13, Roche Ltd, Basel, Switzerland) to NK-cells incubated in the presence of Raji cells (Sigma–Aldrich, Gillingham, UK) for all individuals, as well as in the presence of autologous B-cells for 7 SLE patients and 5 healthy controls above using E:T ratio of 1:1, as well as GolgiStop™ (BD Biosciences, Oxford, UK) for 4 h. Following incubation, anti-CD107a-FITC (REA792, Miltenyi Biotec, Surrey, UK) was added and NK-cells co-stained with anti-CD3 (REA613, Miltenyi Biotec, Surrey, UK), anti-CD56 (AF12-7H3, Miltenyi Biotec, Surrey, UK) and anti-CD16 (REA423, Miltenyi Biotec, Surrey, UK) antibodies. Degranulation activity was measured by the ratio between (percentage CD107a positivity Raji cells only) and (percentage CD107a positivity in rituximab-coated Raji cells) (Supplementary Fig. S3). In terms of reliability, there was a good agreement in ratio degranulation between Raji cells and autologous B-cells when used as target cells, mean difference as assessed by Bland-Altman limit of agreement (LOA), −0.42% (90% CI LOA -1.93 to 1.08). Since the majority of SLE patients had low lymphocyte counts, we continued to perform the functional study using Raji cells as target cells only and presented our results accordingly. Flow cytometry was performed using a Becton Dickinson (BD) LSRII or BD FACSCalibur flow cytometer for data acquisition and using a BD FACSDiva software for data analyses.

### Statistical analyses

Associations between baseline demographic, clinical/laboratory variables and clinical response measures were assessed using linear regression of the 6-month measures, adjusting for corresponding baseline measures for RA; logistic regression was used for SLE outcomes. Complete B-cell depletion post-rituximab for the combined RA and SLE cohort was assessed using logistic regression, without adjustment and following adjusting for age, concomitant DMARD use and baseline plasmablasts. For RA, negative coefficients for the adjusted 2C-DAS28CRP response indicate a favourable outcome.

For three SNPs in two genes (*FCGR2A*, *FCGR2B*) with no reported CNV, associations between the SNPs and outcomes were assessed using standard genotypic tests, whereby individuals homozygous for the common allele served as a reference group. An additive model was also performed. For genes affected by CNV (*FCGR2C*, *FCGR3A*, *FCGR3B*), three analyses were carried out. The first, emulating previous studies that did not take CNV into consideration and assuming 2 gene copies; the second comparing 2 gene CN with deletions (0–1 CN) and duplications (3–4 CN), irrespective of allele carriage; the third based on minor allele CN and total CN. To determine whether the minor allele CN improves on the nested model with total CN, a likelihood ratio test was performed.

Since the RA and SLE cohorts were of mixed ethnicity, we assessed the potential for population stratification by measuring pairwise LD between the relevant functional polymorphisms in the *FCGR* locus for each subgroup. We utilised Haploview to calculate r^2^ LD between biallelic markers in individuals with two copies of *FCGR3A* and *FCGR3B*.

Continuous variables were compared using Mann–Whitney test or Kruskal–Wallis H test, depending on data distribution and number of independent groups for comparison. Spearman's test was used for all correlations. Associations between categorical variables were tested by Fisher's exact test if expected number was ≤5, otherwise chi-squared tests were performed. All statistical analysis was performed using StataMP v.16 (StataCorp College Station, Texas, USA), SPSS v.26 (IBM Corp, Armonk, New York, USA) and GraphPad Prism v.8.3 (GraphPad Prism, La Jolla, California, USA).

### Role of funders

The funders played no role in the study design, data collection, data analysis, interpretation, writing of the report, or the decision of paper submission.

## Results

**Fig. S4:**
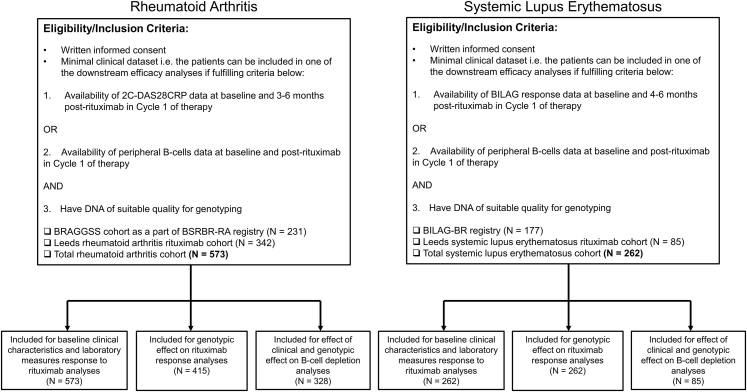


The patient flow chart is illustrated in Supplementary Fig. S4. A total of 835 patients were included in the final analyses and for all Tables and Figures, we have included the sample size for each of the analysis presented to highlight missing clinical data. Some clinical variables were only available for the Leeds cohort, and these are highlighted in the footnotes of Table 1, Table 2. The remaining data were missing at random.

**Table 1:** Baseline clinical characteristics, laboratory measures and association with clinical outcomes and complete B-cell depletion in RA.

Baseline measure or characteristic	Mean (SD) or number (%) positive, N^a^ N = 573	Effect on 2C-DAS28CRP at 6 months: coefficient (SE), *p-value, N*^b^ N = 415	Effect on Complete B-cell depletion: OR (95% CI), *p-value*, N^c^ N = 328
Age at first RTX cycle (effect per 10 years)	58.7 (12.4), 519	−0.06 (0.05), *0.26*, 380	1.03 (1.01–1.05), ***0.01***, 309
Sex (Female)	436 (76%), 556	0.15 (0.15), *0.33*, 415	2.02 (1.16–3.54), ***0.01***, 328
Disease duration (effect per 10 years)	12.43 (10.1), 514	−0.02 (0.06), *0.78*, 377	1.03 (1.01–1.06), ***0.01***, 307
Concomitant DMARDs^d^^,^^e^	233 (80.1%), 291	−0.41 (0.25), *0.09*, 170,	2.09 (1.24–3.52), ***0.01***, 302
Previous TNFi exposure^e^	232 (71.4%), 291	0.49 (0.21), ***0.02***, 170	1.24 (0.75–2.05), *0.40*, 302
No. of previous biologics^e^	1.7 (1.4), 291	0.15 (0.07), ***0.04***, 170	1.09 (0.92–1.28), *0.32*, 302
RF (positive)	367 (81.2%), 452	−0.17 (0.18), *0.35*, 294	0.88 (0.46–1.69), *0.70*, 327
ACPA (positive)	344 (87.9%), 393	0.01 (0.26), *0.98*, 241	0.68 (0.33–1.37), *0.28*, 314
RF or ACPA (positive)	415 (91.5%), 454	−0.33 (0.24), *0.18*, 296	1.25 (0.44–3.52), *0.68*, 328
IgM (g/L)^e^	1.5 (0.9), 330	0.03 (0.10), *0.72*, 177	0.72 (0.55–0.94), ***0.02***, 319
IgA (g/L)^e^	3.3 (1.4), 331	0.07 (0.07), *0.31*, 177	0.82 (0.70–0.95), ***0.01***, 320
IgG (g/L)^e^	12.5 (3.8), 331	−0.04 (0.02), *0.15*, 176	0.93 (0.87–0.98), ***0.01***, 320
Total B-cell counts^e^ (x10^9^/L)	0.13 (0.13), 327	−0.75 (0.80), *0.35*, 172	0.29 (0.05–1.53), *0.15*, 322
Naïve B-cell counts^e^ (x10^9^/L)	0.10 (0.11), 327	−1.57 (0.97), *0.11*, 172	0.34 (0.05–2.54), *0.30*, 322
Memory B-cell counts^e^ (x10^9^/L)	0.03 (0.04), 327	3.35 (2.68), *0.21*, 172	0.03 (0.00–8.48), *0.23*, 322
Plasmablast counts^e^ (x10^9^/L)	0.004 (0.007), 332	10.24 (17.17), *0.55*, 177	0.81 (0.75–0.89), ***<0.001***, 324

The Bolding indicate variables with statistically significant association with the corresponding outcomes to rituximab. All *p-values* should be in *Italic* to separate this from effect on clinical outcomes and number of sample size.

ACPA, anti-cyclic citrullinated peptide antibody; Ig, immunoglobulin; RF, rheumatoid factor; RTX, Rituximab; TNFi, tumour necrosis factor inhibitor.

a Sample size varies in different analyses due to missing clinical data. The N in this column reflects individuals included in analyses for either effect of 2C-DAS28CRP at 6 months and/or complete B-cell depletion.

b Coefficient, standard error (SE) and p*-value* for the effect of each baseline characteristic on the 2-component DAS28 (2C-DAS28CRP) at 6-months. All models were adjusted for the corresponding baseline measure. Negative coefficients for clinical response outcomes indicate a favourable outcome.

c Odds ratio (OR), 95% confidence interval (CI), p-value and number of observations for the effect of the baseline characteristic on complete B-cell depletion at 2 weeks in the Leeds cohort.

d Concomitant disease modifying anti-rheumatic drugs (DMARDs) included hydroxychloroquine, methotrexate and leflunomide.

e Data were only available from the Leeds cohort. All remaining missing data was missing at random.

**Table 2:** Baseline clinical characteristics, laboratory measures and association with clinical outcomes and depletion in SLE.

Baseline measure or characteristic	Mean (SD) or number (%) positive, N^a^ N = 262	Effect on BILAG response (Major or Partial Clinical Response) at 6 months: OR (95% CI), *p-value*, N^b^ N = 262	Effect on BILAG Major Clinical Response at 6 months: OR (95% CI), *p-value*, N^b^ N = 262	Effect on complete B-cell depletion: OR (95% CI), *p-value*, N^b^ N = 85
Age at first RTX cycle (effect per 10 Years)	40 (14), 262	0.81 (0.67–0.98), ***0.03***, 262	0.88 (0.73–1.06), *0.17*, 262	0.91 (0.67–1.24), *0.56*, 85
Sex (Female)	238 (91%), 262	1.55 (0.66–3.66), *0.31*, 262	1.05 (0.43–2.56), *0.91*, 262	0.53 (0.05–6.02), *0.61*, 85
Ethnicity				
Caucasian	161 (61.5%)	1.06 (0.62–1.80), *0.84*, 262^c^	1.18 (0.70–1.98), *0.54*, 262^c^	1.13 (0.45–2.84), *0.80*, 85^c^
South Asian	39 (14.9%)			
Chinese/SE Asian	13 (5.0%)			
Afro-Caribbean	31 (11.8%)			
Mixed/Undisclosed	18 (6.8%)			
Disease Duration at first RTX cycle (effect per year)	8,^6^ 261	0.99 (0.96–1.02), *0.56*, 261	0.99 (0.96–1.03), *0.64*, 261	1.03 (0.96–1.10), *0.45*, 85
Concomitant DMARDs^d^	60 (70.6%), 85	0.69 (0.22–2.14), *0.52*, 85	0.81 (0.31–2.11), *0.66*, 85	0.99 (0.39–2.51), *0.98*, 85
Concomitant anti-malarials,	225 (85.9%), 262	1.32 (0.64–2.72), *0.45*, 262	0.96 (0.46–1.99), *0.91*, 262	1.94 (0.67–5.61), *0.22*, 85
Concomitant oral prednisolone	193 (73.7%), 262	0.80 (0.44–1.46), *0.48*, 262	1.06 (0.59–1.90), *0.84*, 262	1.10 (0.42–2.90), *0.85*, 85
No. positive autoantibodies	1.9 (1.3), 186	1.13 (0.89–1.43), *0.33*, 186	1.02 (0.82–1.28), *0.86*, 186	0.80 (0.57–1.12), *0.19*, 85
anti-Ro	98 (49.2%), 199			
anti-La	38 (19.1%), 199			
anti-Sm	55 (28.4%), 194			
anti-RNP	67 (33.8%), 198			
Anti-dsDNA positive	137 (52.5%), 261	1.33 (0.79–2.25), *0.28*, 261	1.13 (0.68–1.88), *0.65*, 261	0.71 (0.30–1.67), *0.43*, 85
ENA positive	130 (69.9%), 186	1.02 (0.51–2.01), *0.96*, 261	1.05 (0.55–2.02), *0.88*, 261	0.54 (0.21–1.36), *0.19*, 81
Low C3 and/or C4 titre	120 (46%), 261	1.49 (0.88–2.53), *0.14*, 261	1.57 (0.94–2.63), *0.08*, 261	0.35 (0.14–0.88), ***0.03***, 85
Immunoglobulin (g/L)				
IgM	1.33 (1.9), 238	0.90 (0.76–1.07), *0.22*, 238	0.99 (0.86–1.15), *0.93*, 238	1.52 (0.82–2.82), *0.18*, 78
IgA	3.88 (1.9), 238	0.94 (0.78–1.12), *0.49*, 238	0.98 (0.84–1.14), *0.76*, 238	0.70 (0.47–1.06), *0.09*, 78
IgG	16.9 (5.4), 238	0.97 (0.93–1.01), *0.12*, 238	1.00 (0.98–1.01), *0.60*, 238	0.95 (0.88–1.02), *0.13*, 78
ESR (mm/h)	30.4,^27^ 192	0.99 (0.98–1.00), ***0.05***, 192	1.00 (0.99–1.01), *0.88*, 192	0.99 (0.97–1.01), *0.30*, 62
Total B-cell counts (x 10^9^/L)^d^	0.1263 (0.13), 73	1.00 (1.00–1.01), *0.74*, 73	1.00 (1.00–1.01), *0.09*, 73	1.00 (1.00–1.00), 0.79, 73
Naïve B-cell counts (x 10^9^/L)^d^	0.0928 (0.09), 71	1.00 (0.99–1.01), *0.99*, 71	1.00 (1.00–1.01), *0.17*, 71	1.00 (0.99–1.00), *0.54*, 71
Memory B-cell counts (x 10^9^/L)^d^	0.0292 (0.07), 71	1.00 (0.99–1.02), *0.68*, 71	1.02 (0.99–1.04), *0.22*, 71	1.00 (0.99–1.01), *0.71*, 71
Plasmablast counts (x 10^9^/L)^d^	0.0054 (0.01), 71	0.98 (0.90–1.06), *0.60*, 71	0.95 (0.87–1.04), *0.23*, 71	0.88 (0.80–0.98), ***0.02***, 71
Global BILAG score	22 (9.7), 262	1.00 (0.97–1.02), *0.83*, 262	1.00 (0.97–1.03), *0.93*, 262	1.00 (0.96–1.04), *0.96*, 85
SLEDAI-2K score	10.8 (5.7), 262	1.06 (1.00–1.11), ***0.03***, 262	1.02 (0.98–1.07), *0.40*, 262	0.97 (0.90–1.05), *0.46*, 85
Active BILAG domains (A/B Grade)				
General	36 (14.1%)	–	–	–
Mucocutaneous	129 (49.2%)			
Neurology	52 (19.8%)			
Musculoskeletal	121 (46.2%)			
Cardiorespiratory	44 (17.2%)			
Gastrointestinal	18 (6.9%)			
Ophthalmic	9 (3.4%)			
Renal	114 (43.1%)			
Haematology	23 (9.5%)			

The Bolding indicate variables with statistically significant association with the corresponding outcomes to rituximab. All *p-values* should be in *Italic* to separate this from effect on clinical outcomes and number of sample size. BILAG, British Isles Lupus Assessment Group; ENA, extract nuclear antigen; ESR, erythrocyte sedimentation rate; SE, South East; SLEDAI-2K, SLE Disease Activity Index v.2000.

a Sample size varies in different analyses due to missing clinical data. The number (N) in each set of analyses is given.

b OR, 95% CI, p-value and number of observations for the effect of the baseline characteristic on clinical response measures or complete B-cell depletion at 6 weeks. Peripheral blood B-cell depletion data were only available for the Leeds cohort.

c Due to sample size for each ethnicity category, comparison was made between Caucasian (reference) vs Non-Caucasian.

d Data were only available from the Leeds cohort. All remaining missing data was missing at random.

### Association of baseline characteristics with rituximab response and complete B-cell depletion in RA

The baseline characteristics of our RA cohort (n = 573) are described in Table 1.

Previous TNFi exposure (p = 0.02) and number of previous biologics (p = 0.04) were associated with reduced 2C-DAS28CRP response when assessed using linear regression. No clear associations were observed between other salient baseline clinical and serological markers and 6-month 2C-DAS28CRP response, adjusted for corresponding baseline 2C-DAS28CRP.

Complete B-cell depletion 2-weeks after rituximab therapy was achieved in 192/328 (58.5%) patients with data available. Baseline factors associated with increased odds of achieving complete depletion as assessed using logistic regression were older age at baseline (p = 0.01), female (p = 0.02), longer disease duration (p = 0.01), and concomitant DMARD use (p = 0.01). Higher immunoglobulin (IgM, IgA, IgG) levels (p = 0.02, 0.01, 0.01, respectively) and higher plasmablast counts at baseline (p < 0.001) were associated with lower odds of complete depletion (Table 1).

### Association of baseline characteristics with rituximab response and complete depletion in SLE

The baseline characteristics of the SLE cohort (n = 262) are described in Table 2.

At 6-months, 177/262 (67.6%) patients achieved a BILAG response (MCR and/or PCR) and 90/262 (34.4%) achieved a BILAG MCR. Higher SLE Disease Activity Index version 2000 (SLEDAI-2K) score was associated with increased odds of BILAG response at 6-months (p = 0.03), while older age reduced the odds of BILAG response (p = 0.03) when assessed using logistic regression. No other baseline clinical or serological markers were associated with clinical response.

At 6-weeks post-rituximab, 44/85 (51.8%) patients with data available achieved complete B-cell depletion. Low complement (C3 and/or C4) (p = 0.03) and higher plasmablast counts at baseline (p = 0.02) were associated with reduced odds of complete depletion when assessed using logistic regression (Table 2).

### *FCGR2C* QSV assay and functional interpretation of *FCGR2C* genotyping

To supplement the MLPA panels, we developed a *FCGR2C* QSV assay to more accurately determine the *FCGR2C* CN and biologically validated this assay in individuals with different rearrangements of the *FCGR* locus (Fig. 1a). Using samples from treatment-naïve, early RA patients, we observed increased NK-cell CD32 expression with *FCGR2C*-ORF carriage (Mann–Whitney test; p < 0.0001) and in individuals hemizygous for the CNR1 deletion, which carries both *FCGR2C* and *FCGR3B* (Fig. 1b). The MLPA panels used in the current study could not distinguish between classical and non-classical *FCGR2C*-ORF alleles.^21^ A previous study has shown that individuals with non-classical *FCGR2C-ORF* have a frame-shift insertion that leads to a premature STP codon leading to no FcγRIIc expression on the cell surface. Individuals with a hemizygous CNR1 deletion and a *FCGR2C*-STP allele have previously been shown to express FcγRIIb on most NK-cells^21^ (Fig. 1c). Detailed *FCGR2C* SNP and CN data are therefore required to determine whether NK-cells expressing CD32 have an activating (FcγRIIc) or inhibitory (FcγRIIb) FcγR.

### Association of *FCGR* genotype and copy number with rituximab response in RA

**Fig. 2:**
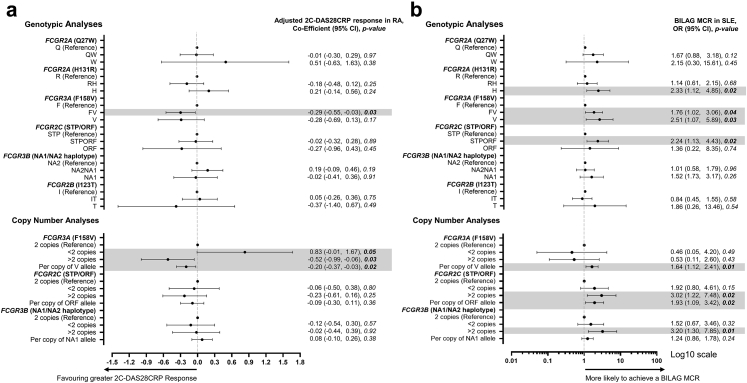
**Association of *FCGR* genotype and copy number with clinical response in RA and SLE.(a)** Co-efficient, 95% confidence intervals (CI) and p-value for the effect of the indicated genotype or copy number on 2C-DAS28CRP response (adjusted for baseline 2C-DAS28CRP) at 6 months post-rituximab, compared with reference genotype. All tests were performed using univariable linear regression; negative coefficients for clinical response outcomes indicate a favourable outcome. **(b)** Odds ratio (OR), 95% CI and p-value for the effect of the indicated genotype or copy number on BILAG Major Clinical Response (MCR) at 6 months post-rituximab, compared with reference genotype. All tests were performed using logistic regression. The x-axis was transformed to log10 scale. For both figures, the dots represent either the co-efficient or the OR and the error bars denote the 95% CI. The vertical broken lines denote lines of no effect.

The *FCGR3A* (rs396991; F158V) variant was first analysed using linear regression at the genotypic level; *FCGR3A*-158V was associated with improved 2C-DAS28CRP response (p = 0.03). Compared to patients with two *FCGR3A* copies (the majority), those with duplications had improved responses (2C-DAS28CRP; p = 0.03). These facets were combined and increased *FCGR3A*-158V CN was associated with improved response (2C-DAS28CRP; p = 0.02) (Fig. 2a; Supplementary Table S6). In a model including both *FCGR3A*-158V CN and *FCGR3A* CN, there was evidence that total *FCGR3A* CN also contributed to response (2C-DAS28CRP; p = 0.04). No associations were observed with other *FCGR* variants. We have provided results from a post-hoc analysis of the 3C-DAS28CRP in Supplementary Table S7 to support future meta-analyses. This provided broadly comparable results.

### Association of *FCGR* genotype and copy number with rituximab response in SLE

To provide cross-disease replication, we performed an equivalent genotypic analysis in SLE using logistic regression (Fig. 2b; Supplementary Table S6). *FCGR2A*-131H homozygosity was associated with an increased odds of BILAG MCR at 6-months (p = 0.02).

Concordant with our RA findings, *FCGR3A* was associated with increased odds of BILAG MCR when analysed at the genotypic level, with *FCGR3A-158V* homozygotes demonstrating a 2.5-fold improved responses (p = 0.03). Carriage of *FCGR3A*-158V was associated with a 1.9-fold (p = 0.02) and a 1.8-fold (p = 0.04) improvement in odds of BILAG response and BILAG MCR, respectively. In contrast to RA, we did not find evidence of an association between *FCGR3A* CN and clinical response, most likely because the majority of patients carried two *FCGR3A* copies (248/262). For each *FCGR3A-*158V allele, there was an increased odds of BILAG MCR at 6-months (p = 0.01).

At the genotypic level, for *FCGR2C*, carriage of the *FCGR2C*-ORF allele was associated with a 2.2-fold improvement in odds of BILAG MCR (p = 0.02). Furthermore, *FCGR2C* duplications had a 3-fold increased odds of BILAG MCR at 6-months (p = 0.02), and a 1.9-fold improved response per *FCGR2C*-ORF copy (p = 0.02). We observed modest linkage disequilibrium between *FCGR3A*-F158V and *FCGR2C-*STP/ORF (r^2^ 0.29) in those with diploid genomes at the *FCGR* locus (Supplementary Table S5). When both the number of copies of *FCGR3A*-158V and number of copies of *FCGR2C*-ORF were included in the model, the association with both genes reduced.

For *FCGR3B*, patients with duplications also had the highest odds of achieving BILAG MCR compared to those with two copies (p = 0.01). The associations with *FCGR2C* and *FCGR3B* CN were not independent as 17/22 subjects with a *FCGR3B* duplication also had a *FCGR2C* duplication indicating a CNR1 duplication in the majority (Fig. 1a). All 22 SLE patients who had *FCGR3B* deletion also had a *FCGR2C* deletion (CNR1) and 21/22 carried the *FCGR2C*-STP allele. There was no association of this rearrangement that would be predicted to be associated with FcγRIIb expression in NK-cells with response, although the power was low.

There were insufficient number of non-Caucasian patients with 2 copies of *FCGR3A*, *FCGR2C* and *FCGR3B* to accurately determine whether this group had different patterns of linkage disequilibrium to the Caucasian population (Supplementary Table S5). A Caucasian-only sensitivity analysis was performed using logistic regression, with broadly similar results, whereby the per allele effect size of MCR was 1.62 (0.97–2.71, p = 0.07) for *FCGR3A*-158V and 1.75 (0.91–3.36, p = 0.09) for *FCGR2C*-ORF (Supplementary Table S8).

### Association of *FCGR* F158V genotype and copy number with complete B-cell depletion

Since peripheral B-cells were analysed using HSFC in the Leeds cohorts only, data from both RA and SLE were combined to increase statistical power (n = 413) and analysed using logistic regression. There was no significant difference in depth of depletion between the disease groups (p = 0.26), although there was a significant difference in age (p = 0.002). The baseline clinical variables associated with depletion in the combined group are shown in Supplementary Table S9. Older age (p = 0.02), female sex (p = 0.04) concomitant DMARDs including hydroxychloroquine (p = 0.003), IgA (p = 0.01), IgG (p = 0.003) and plasmablasts (p < 0.001) were all associated with complete B-cell depletion. A weakly positive correlation was found between baseline IgG and plasmablast counts (Spearman's correlation; r = 0.12; p = 0.02).

**Fig. 3:**
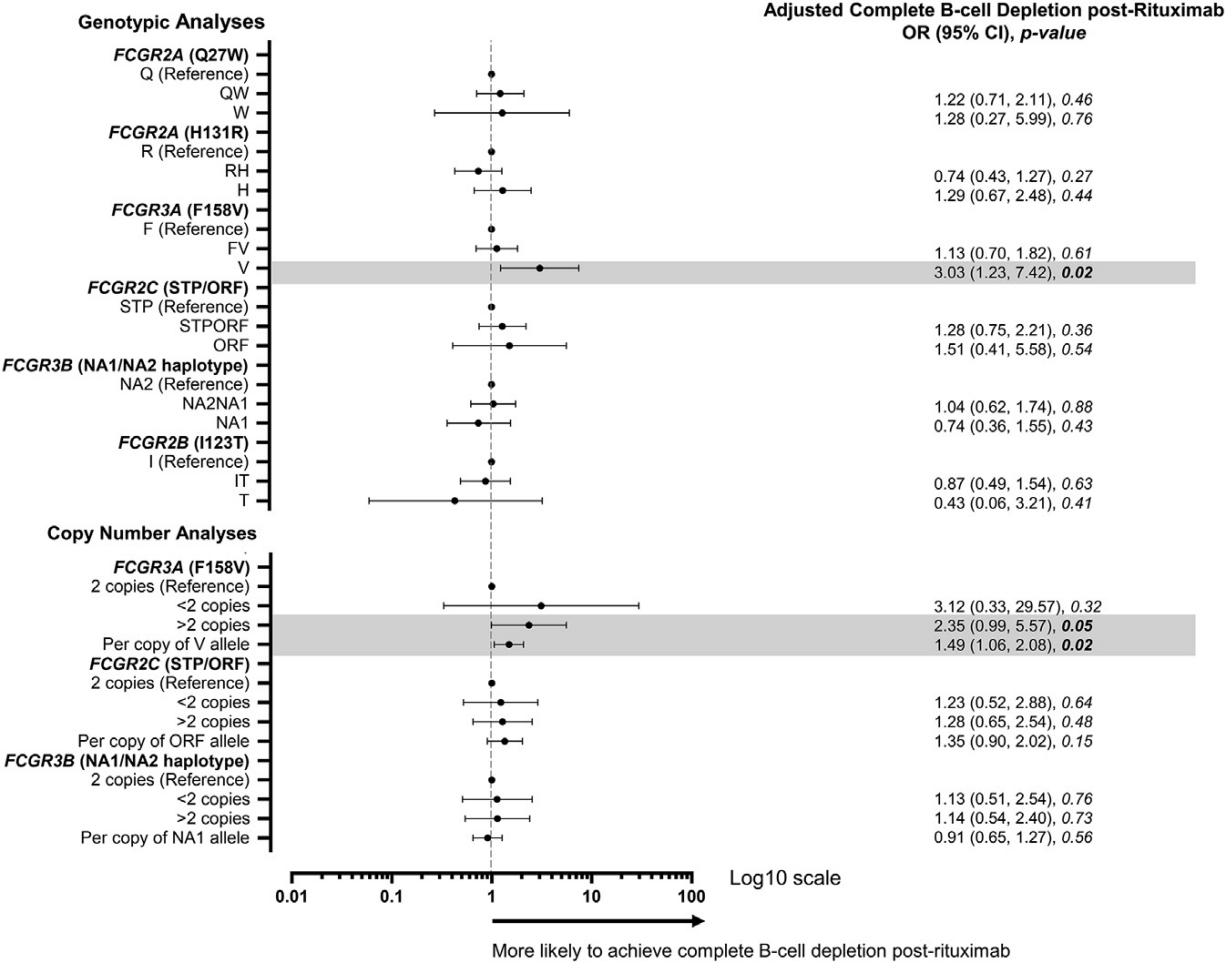
**Association of *FCGR* genotype and copy number with complete B-cell depletion.** Odds ratio (OR), 95% confidence intervals (CI) and p-value for the effect of the indicated genotype or copy number on complete B-cell depletion post-rituximab, compared with reference genotype. The x-axis was transformed to log10 scale. The dots represent the OR and the error bars denote the 95% CI. All tests were performed using logistic regression, adjusted for age, concomitant disease-modifying anti-rheumatic drug use including hydroxychloroquine, and baseline plasmablast count.

Downstream genetic analyses are presented both unadjusted and adjusted for age, concomitant DMARDs, including hydroxychloroquine, and plasmablasts (Fig. 3; Supplementary Table S10). For the CNV-affected genes, increased *FCGR3A*-158V CN was associated with greater odds of B-cell depletion (p = 0.02), in adjusted and unadjusted logistic regression analyses.

### Factors that modulate NK-cell FcγRIIIa expression in the RA disease continuum and SLE

**Fig. 4:**
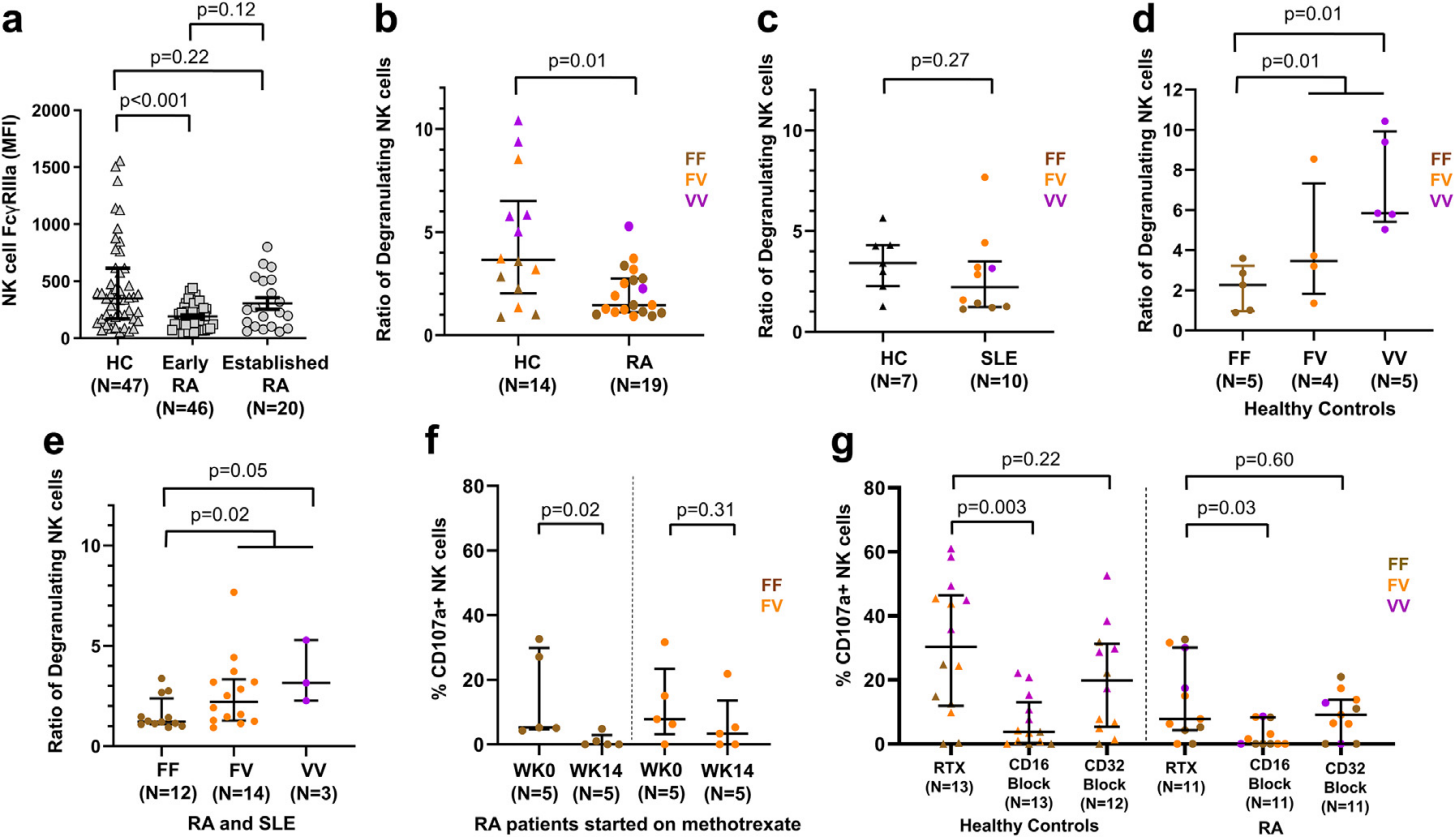
**Effect of disease, genotype and methotrexate on NK-cell degranulation.(a)** Natural Killer (NK)-cell (CD3-CD56+CD16+) FcγRIIIa (CD16; clone 3G8) geometric mean fluorescence intensity (MFI) using flow cytometry for healthy controls (HC) (n = 47), early (symptom onset <1 year and treatment naïve) (n = 46) and established (>2 years) rheumatoid arthritis (RA) (n = 20). Comparison of NK-cell degranulation following incubation with B-cell lineage, Daudi cells between HC (n = 14) and RA (n = 19) **(b)** and Raji cells between HC (n = 7) and systemic lupus erythematosus (SLE) (n = 10) **(c)** using an effector:target (E:T) ratio of 1:1 and rituximab. Ratio of degranulating NK-cells were compared in individuals with two copies of *FCGR3A* between the three *F158V* genotypes in HC (n = 14) **(d)** and RA and SLE combined (n = 29) **(e)**. **(f)** NK-cell degranulation (%CD107a positive NK-cells) was compared before and 14 weeks after RA patients (n = 10) started on methotrexate according to their *FCGR3A* genotype. **(g)** %CD107a positive NK-cells was assessed after incubation with rituximab and subsequent inclusion of CD16 (clone 3G8) and CD32 (clone KB61) blocking antibodies in HC (n = 13) and RA (n = 11). All p-values calculated using non-parametric Mann–Whitney test. Data were summarised as median and the error bars denoted interquartile range.

To disentangle gene and disease-specific effects on NK-cell number and function, we characterised NK-cell FcγRIIIa expression and cellular cytotoxicity *ex vivo*. NK-cell FcγRIIIa expression was measured by flow cytometry in HC (n = 47), early RA (symptom onset <1 year and DMARD-naïve; n = 46) and established RA (diagnosed >2 years and receiving DMARDs; n = 20). FcγRIIIa expression differed between the three groups (Kruskal–Wallis H test; p = 0.01) and was reduced in early (Mann–Whitney test; p < 0.001), but not established RA (Mann–Whitney test; p = 0.22), when compared to HC (Fig. 4a). This differential expression was not secondary to altered ratios of CD56^bright^ to CD56^dim^ NK-cells between RA and HC (Supplementary Fig. S5a). Early RA patients showed lower FcγRIIIa expression on both NK-cell subsets (Mann–Whitney test; both p = 0.02) compared to HC (Supplementary Figs. S5b and S5c). Indeed, there was no difference in FcγRIIIa expression between HC and established SLE patients recruited on the day of their first rituximab infusion (Supplementary Fig. S5d).

To assess factors contributing to the downregulation of NK-cell FcγRIIIa expression in early RA, we examined its relationships with clinical and serological markers using Spearman's correlation test. There were moderate positive correlations between NK-cell FcγRIIIa expression and RF titre (r = 0.38; p = 0.03) and serum IL-6 titre (r = 0.38; p = 0.03), but no significant correlation with CRP (r = 0.14; p = 0.47), ESR (r = 0.16; p = 0.38), DAS28 (r = −0.09; p = 0.69), age (r = −0.02; p = 0.92), nor gender (p = 0.82).

NK-cell FcγRIIIa expression was then examined in relation to the *FCGR3A*-F158V variant in individuals with two *FCGR3A* copies. No significant differences in NK-cell FcγRIIIa expression were demonstrated between *FCGR3A* genotypic groups in either HC (Kruskal–Wallis H test; p = 0.74; Supplementary Fig. S5e), RA (Kruskal–Wallis H test; p = 0.96; Supplementary Fig. S5f) or SLE (Mann–Whitney test; p = 0.21; Supplementary Fig. S5g).

### Disease, genotype and DMARDs impact efficiency of NK-cell-mediated ADCC

NK-cell degranulation (CD107a staining) following exposure to rituximab-coated B lineage cell lines was used as a surrogate of ADCC in individuals with two *FCGR3A* copies. Lysosomal-associated membrane protein-1 (LAMP1 or CD107a) is an established marker of NK-cell degranulation and has been shown to be highly correlated with NK-cell-mediated lysis of chromium labelled target cells.^50^

NK-cell degranulation was significantly decreased in RA (Mann–Whitney test; p = 0.01; Fig. 4b), but not SLE (Mann–Whitney test; p = 0.27; Fig. 4c) compared with HC. *FCGR3A* genotype was associated with rituximab-induced NK-cell degranulation in HC, RA and SLE, whereby *FCGR3A-*158V carriage and *FCGR3A-*158V homozygosity were associated with greater degranulation in HC (Mann–Whitney test; both p = 0.01; Fig. 4d), RA and SLE combined (Mann–Whitney test; p = 0.02, 0.05; Fig. 4e), respectively, compared to *FCGR3A-*138FF.

Reduced degranulation in RA compared with HC, prompted us to explore the impact of DMARDs on NK-cell degranulation *ex vivo* (Fig. 4f). A significant reduction in the % CD107+ NK-cells was observed after 14 weeks of methotrexate in RA, compared to baseline, with the most marked reduction occurring in *FCGR3A-*158F homozygotes (Mann–Whitney test; p = 0.02).

Inclusion of a CD16 blocking antibody inhibited rituximab-induced NK-cell degranulation in HC (Mann–Whitney test; p = 0.003) and RA (Mann–Whitney test; p = 0.03, Fig. 4g), supporting a major role for FcγRIIIa in NK-cell-mediated ADCC *ex vivo*. No significant reduction in NK-cell degranulation was observed with CD32 blockade in HC or RA (Mann–Whitney test; p = 0.22, p = 0.60; Fig. 4g). None of these individuals had CNR1 deletion (FcγRIIb expression) and there was no clear association with *FCGR2C* genotype.

### Higher expression of FcγRIIIa is associated with greater response and depletion *in vivo* independent of NK-cell number

**Fig. 5:**
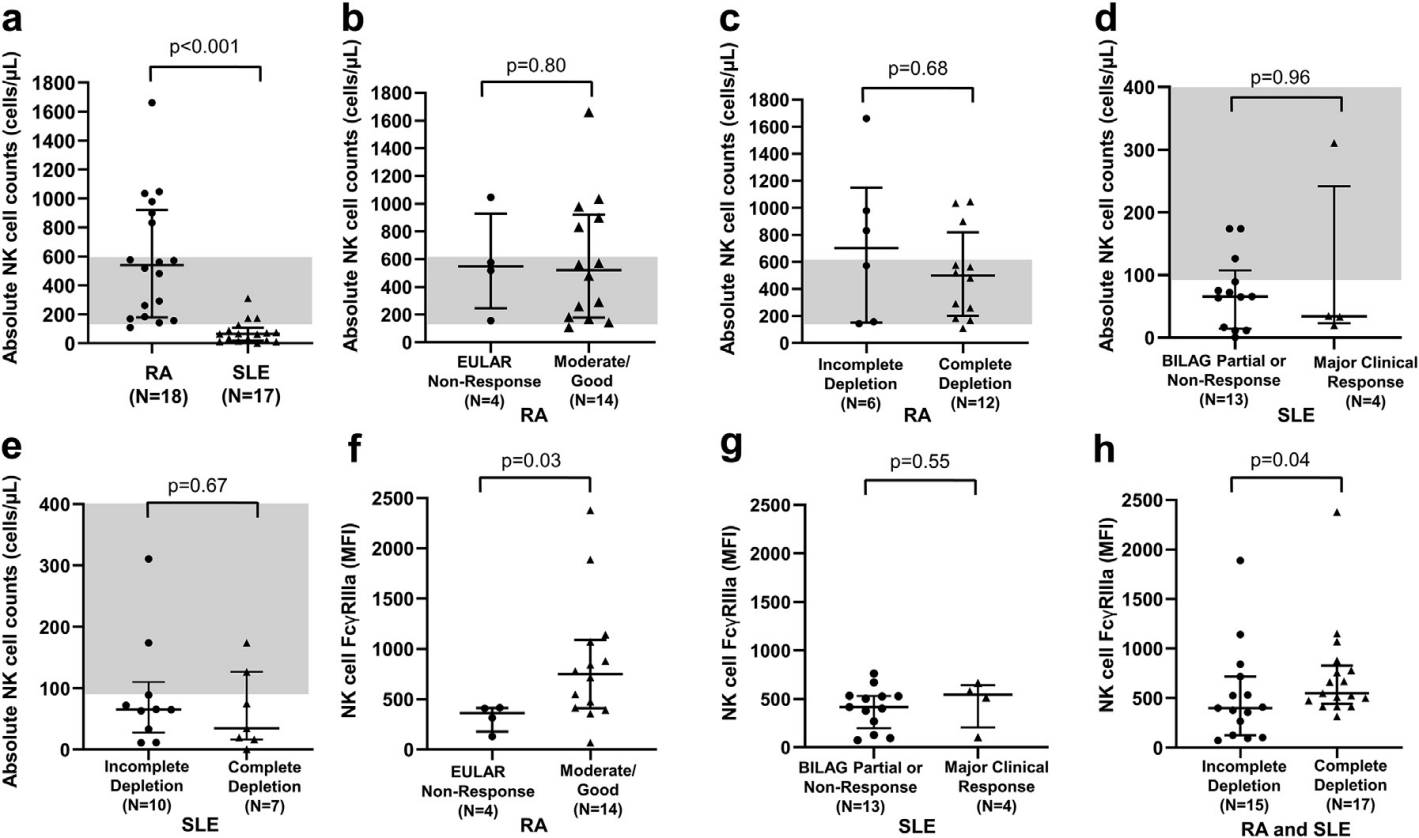
**Peripheral blood NK-cell abundance and FcγRIIIa expression in rituximab-treated RA and SLE patients.** Comparison of absolute natural killer (NK)-cell (CD3-CD56+) counts between **(a)** rituximab-treated rheumatoid arthritis (RA) (n = 18) and systemic lupus erythematosus (SLE) (n = 17) patients; **(b)** rituximab-treated RA patients exhibiting no European League Against Rheumatism (EULAR) clinical response (n = 4) and moderate/good clinical response (n = 14); **(c)** rituximab-treated RA patients exhibiting incomplete (n = 6) and complete B-cell depletion (n = 12); **(d)** rituximab-treated SLE patients exhibiting British Isles Lupus Assessment Group (BILAG) partial clinical response or no clinical response (n = 13) and major clinical response (n = 4); and **(e)** rituximab-treated SLE patients with incomplete (n = 10) and complete B-cell depletion (n = 7). The shaded grey areas represent adult reference ranges of the absolute NK-cell counts (90–600 cells/μL). Expression of FcγRIIIa (CD16; clone 3G8) on NK-cells of **(f)** RA patients exhibiting EULAR non-response (n = 4) and moderate/good clinical response (n = 14) to rituximab; **(g)** SLE patients exhibiting BILAG partial clinical response/non-response (n = 13) and major clinical response (n = 4) to rituximab; and **(h)** RA and SLE patients exhibiting incomplete (n = 15) and complete B-cell depletion (n = 17) in response to rituximab. All p-values calculated using non-parametric Mann–Whitney test. Data were summarised as median and the error bars denoted interquartile range.

Both frequency of NK-cells (CD3-CD56+) and absolute NK-cell number were lower in SLE than RA (Mann–Whitney test; both p < 0.001; Supplementary Figs. S5h and S5a respectively). Due to small sample size in functional study, we dichotomised clinical response in RA into EULAR good-to-moderate (defined as improvement of at least 0.6 point to DAS28CRP≤5.1) vs non-response. There was no evidence for an association between NK-cell number and EULAR response (Mann–Whitney test; p = 0.80; Fig. 5b) or complete B-cell depletion (Mann–Whitney test; p = 0.68; Fig. 5c) in RA, nor BILAG MCR (Mann–Whitney test; p = 0.96; Fig. 5d) or complete B-cell depletion (Mann–Whitney test; p = 0.67; Fig. 5e) in SLE.

We demonstrated a positive association between NK-cell FcγRIIIa expression at rituximab initiation and EULAR good-to-moderate response at 6-months in RA (n = 18, Mann–Whitney test; p = 0.03; Fig. 5f), but not BILAG MCR in SLE (n = 17, Mann–Whitney test; p = 0.55, Fig. 5g). Patients with complete depletion had higher NK-cell FcγRIIIa expression at rituximab initiation than those with incomplete depletion in RA and SLE (Mann–Whiteny test; p = 0.04: Fig. 5h).

## Discussion

We report the largest study, to date, of quantitative and qualitative functional variants at the *FCGR* genetic locus in well-characterised RA and SLE cohorts, including subgroups documenting peripheral blood B-cell subset depletion. Our cohorts are representative of patients with RA and SLE receiving rituximab in the UK and the salient clinical characteristics (age, gender distribution, disease duration and disease activity scores) of our cohorts are comparable to real-world data of rituximab-treated patients from registries in Europe.^51^, ^52^, ^53^, ^54^ We provide consistent evidence that FcγRIIIa is the major FcγR associated with both clinical and biological (depth of B-cell depletion) response to rituximab in two autoimmune diseases. More specifically, increased copies of the higher affinity *FCGR3A*-158V allele, was associated with improved response. Irrespective of *FCGR3A* genotype, we observed that increased *FCGR3A* CN was associated with a better response in RA. In *ex vivo* studies, we demonstrated *FCGR3A* genotype was associated with NK-cell-mediated degranulation, and increased NK-cell FcγRIIIa expression was associated with improved clinical response and depletion *in vivo*.

No consistent association signals were observed with other low affinity *FCGR* genes, suggesting FcγRIIIa is the most important FcγR contributing to rituximab response. Increased NK-cell-mediated degranulation was observed in *FCGR3A*-158V homozygotes, irrespective of disease, which combined with the association between FcγRIIIa expression on NK-cells and response, supports ADCC being a major biological mechanism. This does not preclude FcγRIIIa-mediated clearance of rituximab-opsonised B-cells by tissue macrophages or the reticuloendothelial system by ADCP.^55^ Our data also reveal potential disease or inflammation-specific factors that may impair ADCC, e.g. the reduced NK-cell degranulation and FcγRIIIa expression observed in early RA. Furthermore, we demonstrated reduced ADCC in RA patients after 14 weeks of methotrexate therapy, suggesting medication used during rituximab treatment may modulate NK-cell-mediated effector mechanisms. Further studies are required to understand how NK-cell function can be optimised at the time of rituximab treatment to improve clinical outcomes.

In SLE, higher *FCGR2C*-ORF CN and *FCGR2C* duplications were also associated with BILAG MCR at 6-months. MLPA panels were supplemented with sequencing-based *FCGR2C* genotyping methods, to aid genotype interpretation. We observed NK-cell CD32 expression broadly correlated with *FCGR2C*-ORF carriage, and also confirmed high CD32 expression in individuals with CNR1 deletion (simultaneous *FCGR2C* and *FCGR3B* deletion), previously shown to lead to NK-cell FcγRIIb expression. This rearrangement was observed in 4.3% RA and 5.9% SLE individuals of our cohort, with no evidence it impacted on response.

In RA, we have presented data on the more recently published 2C-DAS28CRP score^45^ that includes revised weighting of CRP and SJC to more closely reflect the ultrasound-detected synovitis and radiographic progression; an outcome measure we have proposed as the RA disease activity measure of choice for genetic and biomarker studies.^44^ In SLE, we used a BILAG-based endpoint rather than SLEDAI based. BILAG is better for biomarker studies because, unlike the SLEDAI, it allows partial improvement and does not include serological components, a serious confounder in studies of a B-cell-targeted therapy. Consistent with our previous studies, we included plasmablasts in our definition of total B-cell depletion. Whilst plasmablasts do not express CD20, their persistence in circulation have been shown to reflect B-cell activity outside the circulation, such as in lymphoid tissues or ectopic germinal centres found in inflamed tissues.^56^, ^57^, ^58^ Our previous work has shown that plasmablast depletion can reflect the dose of rituximab administered as well as the use of combination immunosuppressants, as well as predicting subsequent clinical response to rituximab.^5^^,^^6^^,^^8^^,^^59^^,^^60^ Further, recent analyses of Obinutuzumab in clinical trials in lupus nephritis have also shown an association between plasmablast depletion and clinical response.^61^

Our results have implications for future clinical trial design and development of more effective B-cell depletion strategies. Firstly, patients with increased *FCGR3A*-158V CN may respond to lower rituximab doses during repeat cycles, reducing complications, such as neutropaenia and infections secondary to hypogammaglobulinaemia that develops with repeated courses of rituximab therapy. Secondly, confirmation that FcγRIIIa is the major FcγR contributing to clinical response is of value to therapeutic antibody design and highlights the need for next generation CD20 therapeutics to show equivalent ADCC potency in individuals with both FcγRIIIa-158F/V allotypes. There are now several therapeutics at different stages in development with modified Fc regions to enhance ADCC. Control of fucosylation during manufacture of the FDA-approved Obinutuzumab leads to increased ADCC.^62^ This type 2 CD20 mAb binds to a different CD20 epitope, which results in reduced CDC. It is licenced for subgroups of patients with chronic lymphocytic leukaemia, follicular lymphoma and multiple sclerosis and is being investigated in a phase III trial of lupus nephritis [NCT04221477]. Other approaches to enhancing Fc effector functions include point mutations to the CH2 and CH3 domains (e.g. margetuximab). Thirdly, further consideration needs to be given for factors that upregulate of NK-cell FcγRIIIa expression and function at the time of treatment. Finally, there are also implications for quality control of biosimilar rituximab, where significant batch–batch differences in ADCC are recognised that may disproportionally affect *FCGR3A*-138F homozygotes.^63^ Our data may also be relevant for other cell depleting mAb based with an IgG1-Fc.

The study has some limitations. Some data were missing, which is inevitable when large observation cohorts are utilised. Most notably, B-cell data were only available for patients treated in Leeds. We have explored our data and are unable to find any patterns for the remaining missing data that can be explained according to cohort, period of enrolment e.g. prior to approval for RA in the UK in 2007 vs post-approval or other variables that may have biased our results. We therefore conclude that our data is missing at random. We did not impute missing data to aid future meta-analyses. Next, the only covariates that were significantly associated with 6-month 2C-DAS28CRP response in RA were exposure to previous TNFi and number of previous biologics. These data were only available in the Leeds cohorts. Hence, we elected not to adjust as this would have significantly reduced our sample size. In SLE, we similarly did not see any covariates with consistent associations with both BILAG response and BILAG MCR and for simplicity and ease of future meta-analyses elected to present unadjusted data. We presented nominal p-values without taking into account the number of analyses conducted, and p-values must be interpreted bearing this in mind. The strength of evidence for *FCGR3A* lies in the consistent direction of genetic association in two diseases and through the *ex vivo* and *in vivo* studies. Different experimental protocols (e.g. heparinised whole blood samples vs fresh PBMCs) were used for the flow cytometry and degranulation assays in RA and SLE, which precluded direct comparison of these two disease cohorts.

In conclusion, an ADCC-enhancing quantitative *FCGR3A* variant was associated with clinical response and complete B-cell depletion in rituximab-treated RA and SLE patients. These findings were supported by mechanistic studies demonstrating the impact of FcγRIIIa expression, genotype, disease status and medication on NK-cell-mediated cytotoxicity. These results elucidate one mechanism of impaired rituximab responses, may guide development of more effective B-cell targeted strategies and emphasise the importance of ensuring the next generation of therapeutics bind with equivalent affinity to both FcγRIIIa allotypes.

## Contributors

All authors met the authorship criteria. Conceptualisation: JIR, MYMY, EMV, AWM; Methodology: JIR, MYMY, JHB, EMV, AWM; Data Collection and Resources: DLM, LL, ACR, MHB, DP, HJC, JDI, INB, PE, AB, TJV; Performing Experiments: JIR, MYMY, DW, MM, YES; Data Analysis: JIR, MYMY, VD, DW, MM, JHB, JT, AWM; Writing Original Draft: JIR, MYMY, EMV, AWM. JIR, MYMY, JHB and AWM have verified the underlying data. All authors have contributed in revising the manuscript critically for important intellectual content, approved the submitted version and agreed to be accountable for all aspects of the work in ensuring that questions related to the accuracy or integrity of any part of the work are appropriately investigated and resolved.

## Data sharing statement

All data associated with this study are available in the main text or the online supplementary materials. Upon a justifiable request, the share of de-identified data are available from the corresponding author.

## Declaration of interests

Prof Bruce has received research grants from 10.13039/100004330GSK, consulting fees from GSK, 10.13039/100011110UCB, 10.13039/100004312Eli Lilly & Co, 10.13039/100009689BMS, Aurinia, IL-TOO and 10.13039/100004325AstraZeneca and speaker fees from 10.13039/100004325AstraZeneca, GSK and 10.13039/100011110UCB within the last 3 years. Dr Vital has received honoraria and consulting fees from 10.13039/100004337Roche within the last 3 years. All other authors declare no competing interest related to the work described in this manuscript.

## Figures and Tables

**Figure:**
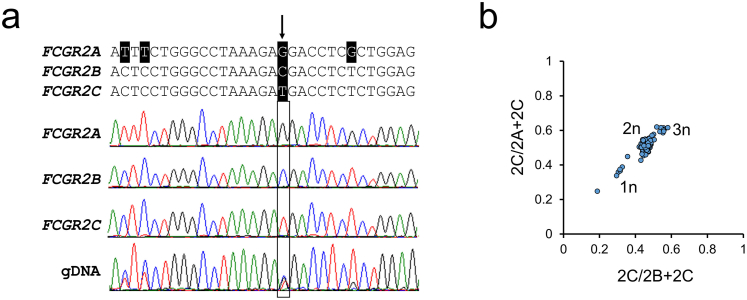
Fig. S1

**Figure:**
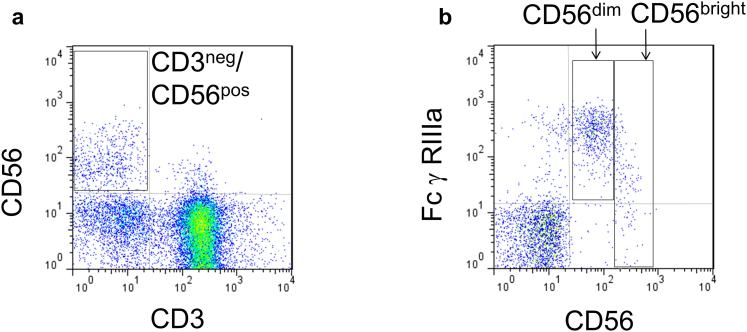
Fig. S2

**Fig. S5:**