# Supplementary material for: Comprehensive genetic and functional analyses of Fc gamma receptors influence on response to rituximab therapy for autoimmunity
Source: eBioMedicine. 2022 Nov 11;86:104343. doi: 10.1016/j.ebiom.2022.104343 (PMC9663864; doi:10.1016/j.ebiom.2022.104343)
Supplement: Supplementary Tables S1–S10 [file mmc1.docx]

**Supplementary Table 1. Oligonucleotide primer sequences used to sequence *FCGR2* genes and a *FCGR2C* QSV assay**

| Gene | Forward | Reverse | Amplicon length (bp) |
| --- | --- | --- | --- |
| *FCGR2A* | dGTGAGCATTTTAGTACCAGTTGCTTTGAC | dCCTTTAACAATTCCCCTCTTTTTGTCATCCACTC | 21,264 |
| *FCGR2B* | dCTCCACAGGTTACTCGTTTCTACCTTATCTTAC | dCCCAGAAAGAATCACTTTTAATGTGCTGG | 16,660 |
| *FCGR2C* | dCTCCACAGGTTACTCGTTTCTACCTTATCTTAC | dCCTTTAACAATTCCCCTCTTTTTGTCATCCACTC | 20,371 |
| *FCGR2A, B & C* | dAGTTCAGCTGGGAGCCAGGGA | dGCCTCAGTCTTACAGCCCCTA (QSV seq primer) | 279 |

**Supplementary Table 2.** ***FCGR* genotype frequencies and Hardy Weinberg equilibrium in the RA and SLE cohorts**

|  |  | **RA (n=611)** | **%** | **HWP^1^** | **SLE (n=594)** | **%** | **HWP^1^** |
| --- | --- | --- | --- | --- | --- | --- | --- |
| ***FCGR2A* Q27W** | QQ | 465 | 76.1 | 0.99 | 458 | 77.1 | 0.33 |
|  | QW | 136 | 22.3 |  | 130 | 21.9 |  |
|  | WW | 10 | 1.6 |  | 6 | 1.0 |  |
|  | Fail/unclear | 0 | 0.0 |  | 0 | 0.0 |  |
| ***FCGR2A* H131R** | HH | 152 | 24.9 | 0.84 | 140 | 23.6 | 0.49 |
|  | HR | 303 | 49.6 |  | 288 | 48.5 |  |
|  | RR | 156 | 25.5 |  | 166 | 27.9 |  |
|  | Fail/unclear | 0 | 0.0 |  | 0 | 0.0 |  |
| ***FCGR3A* F158V** | FF | 243 | 39.8 | 0.75 | 232 | 39.1 | 0.73 |
|  | FV | 247 | 40.4 |  | 245 | 41.2 |  |
|  | VV | 59 | 9.7 |  | 69 | 11.6 |  |
|  | F | 8 | 1.3 |  | 6 | 1.0 |  |
|  | V | 4 | 0.7 |  | 3 | 0.5 |  |
|  | FFF | 4 | 0.7 |  | 13 | 2.2 |  |
|  | FFV | 26 | 4.3 |  | 19 | 3.2 |  |
|  | FVV | 14 | 2.3 |  | 3 | 0.5 |  |
|  | VVV | 5 | 0.8 |  | 1 | 0.2 |  |
|  | FFFV | 0 | 0.0 |  | 1 | 0.2 |  |
|  | FFVV | 1 | 0.2 |  | 0 | 0.0 |  |
|  | Fail/Unclear | 0 | 0.0 |  | 2 | 0.3 |  |
| ***FCGR2C* X57Q** | ORF/ORF | 11 | 1.8 | 0.62 | 9 | 1.5 | 0.70 |
| **(classical STP/ORF)** | STP/ORF | 111 | 18.2 |  | 99 | 16.7 |  |
|  | STP/STP | 335 | 54.8 |  | 317 | 53.4 |  |
|  | del | 0 | 0.0 |  | 2 | 0.3 |  |
|  | ORF | 5 | 0.8 |  | 8 | 1.3 |  |
|  | STP | 42 | 6.9 |  | 60 | 10.1 |  |
|  | ORF/ORF/ORF | 3 | 0.5 |  | 3 | 0.5 |  |
|  | STP/ORF/ORF | 17 | 2.8 |  | 14 | 2.4 |  |
|  | STP/STP/ORF | 10 | 1.6 |  | 17 | 2.9 |  |
|  | STP/STP/STP | 35 | 5.7 |  | 55 | 9.3 |  |
|  | ORF/ORF/ORF/ORF | 1 | 0.2 |  | 0 | 0.0 |  |
|  | STP/ORF/ORF/ORF | 0 | 0.0 |  | 1 | 0.2 |  |
|  | STP/STP/ORF/ORF | 0 | 0.0 |  | 2 | 0.3 |  |
|  | STP/STP/STP/ORF | 2 | 0.3 |  | 2 | 0.3 |  |
|  | STP/STP/STP/STP | 6 | 1.0 |  | 4 | 0.7 |  |
|  | Fail/Unclear | 33 | 5.4 |  | 1 | 0.2 |  |
| ***FCGR3B* NA1/NA2/SH** | NA1/NA1 | 63 | 10.3 | 0.16 | 72 | 12.1 | 0.05 |
|  | NA1/NA2 | 201 | 32.9 |  | 184 | 31.0 |  |
|  | NA2/NA2 | 213 | 34.9 |  | 176 | 29.6 |  |
|  | NA1/SH | 9 | 1.5 |  | 9 | 1.5 |  |
|  | NA2/SH | 3 | 0.5 |  | 7 | 1.2 |  |
|  | SH/SH | 1 | 0.2 |  | 2 | 0.3 |  |
|  | del | 0 | 0.0 |  | 2 | 0.3 |  |
|  | NA1 | 17 | 2.8 |  | 29 | 4.9 |  |
|  | NA2 | 37 | 6.1 |  | 33 | 5.6 |  |
|  | SH | 0 | 0.0 |  | 4 | 0.7 |  |
|  | NA1/NA1/NA1 | 0 | 0.0 |  | 1 | 0.2 |  |
|  | NA1/NA1/NA2 | 14 | 2.3 |  | 21 | 3.5 |  |
|  | NA1/NA1/SH | 0 | 0.0 |  | 4 | 0.7 |  |
|  | NA1/NA2/NA2 | 18 | 2.9 |  | 22 | 3.7 |  |
|  | NA1/NA2/SH | 0 | 0.0 |  | 10 | 1.7 |  |
|  | NA1/SH/SH | 5 | 0.8 |  | 5 | 0.8 |  |
|  | NA2/NA2/NA2 | 1 | 0.2 |  | 4 | 0.7 |  |
|  | NA2/NA2/SH | 0 | 0.0 |  | 2 | 0.3 |  |
|  | NA1/NA1/NA2/NA2 | 0 | 0.0 |  | 1 | 0.2 |  |
|  | NA1/NA1/NA2/SH | 0 | 0.0 |  | 2 | 0.3 |  |
|  | NA1/NA2/NA2/NA2 | 0 | 0.0 |  | 1 | 0.2 |  |
|  | NA2/NA2/NA2/SH | 0 | 0.0 |  | 2 | 0.3 |  |
|  | Fail/Unclear | 29 | 4.7 |  | 1 | 0.2 |  |
| ***FCGR2B* I232T** | II | 475 | 77.7 | 0.98 | 442 | 74.4 | 0.50 |
|  | IT | 124 | 20.3 |  | 143 | 24.1 |  |
|  | TT | 8 | 1.3 |  | 9 | 1.5 |  |
|  | Fail/Unclear | 4 | 0.7 |  | 0 | 0.0 |  |

^1^ Hardy Weinberg equilibrium P value, using diploid genotypes only (shaded rows).

^2^ *FCGR2C* quantitative genotypes were generated using the gene copy number derived from our *FCGR2C* QSV assay as an independent measure.

**Supplementary Table 3. *FCGR3A*, *FCGR2C* and *FCGR3B* gene copy number frequencies in RA and SLE cohorts**

|  |  | **RA** | | **SLE** | | |
| --- | --- | --- | --- | --- | --- | --- |
|  | **Copy number** | **n** | **%** | **n** | **%** |  |
| ***FCGR3A*** | 1 | 12 | 2.0 | 9 | 1.5 | |
|  | 2 | 548 | 89.7 | 547 | 92.2 | |
|  | 3 | 50 | 8.2 | 36 | 6.1 | |
|  | 4 | 1 | 0.2 | 1 | 0.2 | |
| ***FCGR2C*** | 0 | 0 | 0.0 | 2 | 0.3 | |
|  | 1 | 47 | 8.1 | 68 | 11.4 | |
|  | 2 | 457 | 79.1 | 425 | 71.5 | |
|  | 3 | 65 | 11.2 | 90 | 15.2 | |
|  | 4 | 9 | 1.6 | 9 | 1.5 | |
| ***FCGR3B*** | 0 | 0 | 0.0 | 2 | 0.3 | |
|  | 1 | 54 | 8.8 | 66 | 11.1 | |
|  | 2 | 497 | 81.3 | 453 | 76.3 | |
|  | 3 | 53 | 8.7 | 67 | 11.3 | |
|  | 4 | 7 | 1.1 | 6 | 1.0 | |

^1^ *FCGR2C* gene copy number was determined using our QSV assay to complement the limited number of *FCGR2C* specific probes on the B2 version of the P110 and P111 MLPA panels.

**Supplementary Table 4. Copy number region loss and gain frequencies for RA and SLE cohorts**

| **CNR event** | **MATURA** | **BILAG-BR** |
| --- | --- | --- |
| CNR1 gain | 45 (3.7%) | 77 (6.5%) |
| CNR1 loss | 43 (3.5%) | 64 (5.4%) |
| CNR2 gain | 23 (1.9%) | 32 (2.7%) |
| CNR2 loss | 4 (0.3%) | 6 (0.5%) |
| CNR1&2 gain | 4 (0.3%) | 4 (0.3%) |
| CNR1&2 loss | 0 | 0 |

**Supplementary Table 5. Pairwise linkage disequilibrium (r^2^) between biallelic diploid SNP- based markers in the *FCGR* locus in 127 confirmed British Caucasian SLE participants restricted to individuals with two copies of *FCGR3A*, *FCGR2C* and *FCGR3B***

|  | ***FCGR2A* Q27W** | ***FCGR2A* H131R** | ***FCGR3A* F158V** | ***FCGR2C* cSTP/ORF** | ***FCGR3B* NA1/NA2** | ***FCGR2B* I232T** |
| --- | --- | --- | --- | --- | --- | --- |
| ***FCGR2A* Q27W** |  | 0.18 | 0.19 | 0.61 | 0.01 | 0.00 |
| ***FCGR2A* H131R** |  |  | 0.18 | 0.08 | 0.02 | 0.00 |
| ***FCGR3A* F158V** |  |  |  | 0.29 | 0.14 | 0.00 |
| ***FCGR2C* cSTP/ORF** |  |  |  |  | 0.03 | 0.01 |
| ***FCGR3B* NA1/NA2** |  |  |  |  |  | 0.03 |

**Supplementary Table 6. Effect of *FCGR* genotype and copy number on clinical response to rituximab in RA and SLE**

| ***Gene^1^*** | **Genotypes/**  **Copy Number** | **Rheumatoid Arthritis** | **Systemic Lupus Erythematosus** | |
| --- | --- | --- | --- | --- |
|  |  | **Effect on 2C-DAS28CRP at 6-month: coefficient (SE)^2^, *p-value*, N** | **BILAG Response (Major or Partial) at 6 months: OR (95% CI)^3^, *p-value*, N** | **BILAG Major Clinical Response at 6 months: OR (95% CI)^3^, *p-value*, N** |
| **Genotypic Analyses** | | | | |
| *FCGR2A*  (Q27W) | Q  (Ref) | -  318 | -  208 | -  208 |
|  | QW | -0.01 (0.15), *0.97*, 92 | 2.01 (0.95 - 4.27), *0.07*, 48 | 1.67 (0.88 - 3.18), *0.12*, 48 |
|  | W | 0.51 (0.57), *0.38*, 5 | 0.53 (0.07 - 3.84), *0.53*, 4 | 2.15 (0.30 - 15.61), *0.45*, 4 |
|  | Additive model | 0.05 (0.14), 0.74, 415 | 1.47 (0.79 – 2.75), *0.23*, 260 | 1.61 (0.93 – 2.81), *0.09*, 260 |
| *FCGR2A*  (H131R) | R  (Ref) | -  103 | -  70 | -  70 |
|  | RH | -0.18 (0.15), *0.25*, 212 | 0.74 (0.40 - 1.38), *0.35*, 134 | 1.14 (0.61 - 2.15), *0.68*, 134 |
|  | H | 0.21 (0.18), *0.24*, 100 | 1.23 (0.56 - 2.68), *0.60*, 58 | 2.33 (1.12 - 4.85), ***0.02***, 58 |
|  | Additive model | 0.10 (0.09), 0.25, 415 | 1.08 (0.74 – 1.56), *0.69*, 262 | 1.53 (1.06 – 2.22), ***0.03***, 262 |
| *FCGR3A^4^* (F158V) | F  (Ref) | -  175 | -  126 | -  126 |
|  | FV | -0.29 (0.13), ***0.03***, 193 | 1.93 (1.10 - 3.39), ***0.02***, 109 | 1.76 (1.02 - 3.06), ***0.04***, 109 |
|  | V | -0.28 (0.21), *0.17*, 47 | 1.27 (0.53 - 3.06), *0.59*, 27 | 2.51 (1.07 - 5.89), ***0.03***, 27 |
|  | Additive model | -0.19 (0.09). **0.04**, 415 | 1.38 (0.92 – 2.06), *0.12*, 262 | 1.64 (1.12 – 2.41), ***0.01***, 262 |
| *FCGR2C*^4^  (STP/ORF) | STP  (Ref) | -  289 | -  173 | -  173 |
|  | STPORF | -0.02 (0.15), *0.89*, 95 | 2.20 (0.96 - 5.05), *0.06*, 42 | 2.24 (1.13 - 4.43), ***0.02***, 42 |
|  | ORF | -0.27 (0.35), *0.45*, 14 | 0.78 (0.13 - 4.78), *0.79*, 5 | 1.36 (0.22 - 8.35), *0.74*, 5 |
|  | Additive model | -0.07 (0.12), 0.57, 398 | 1.55 (0.80 – 3.00), *0.19*, 220 | 1.77 (1.01 – 3.13), ***0.05***, 220 |
| *FCGR3B*^4^  (NA1/NA2 haplotype) | NA2  (Ref) | -  172 | -  106 | -  106 |
|  | NA2NA1 | 0.19 (0.14), *0.19*, 166 | 1.03 (0.58 - 1.81), *0.93*, 111 | 1.01 (0.58 - 1.79), *0.96*, 111 |
|  | NA1 | -0.02 (0.19), *0.91*, 58 | 1.23 (0.56 - 2.69), *0.60*, 42 | 1.52 (0.73 - 3.17), *0.26*, 42 |
|  | Additive model | 0.04 (0.09), 0.69, 396 | 1.09 (0.76 – 1.58), *0.64*, 259 | 1.19 (0.83 – 1.70), *0.34*, 259 |
| *FCGR2B*  (I123T) | I  (Ref) | -  327 | -  197 | -  197 |
|  | IT | 0.05 (0.16), *0.75*, 80 | 0.68 (0.37 - 1.23), *0.20,* 61 | 0.84 (0.45 - 1.55), *0.5*8, 61 |
|  | T | -0.37 (0.53), *0.49*, 6 | 1.31 (0.13 - 12.89), *0.82*, 4 | 1.86 (0.26 - 13.46), *0.54*, 4 |
|  | Additive model | -0.01 (0.14), 0.95, 413 | 0.76 (0.45 – 1.30), *0.32*, 262 | 0.95 (0.55 – 1.63), *0.85*, 262 |
| **Copy Number Analyses** | | | | |
| *FCGR3A* (F158V) | 2 copies (Ref) | -  375 | -  248 | -  248 |
|  | <2 copies | 0.83 (0.43), ***0.05****,* 9 | See Note^5^, 5 | 0.46 (0.05 – 4.20), *0.49*, 5 |
|  | >2 copies | -0.52 (0.24), ***0.03****,* 31 | 0.61 (0.16 – 2.32), *0.47*, 9 | 0.53 (0.11 – 2.60), *0.43*, 9 |
|  | Per copy of V allele | -0.20 (0.09), ***0.02****,* 415 | 1.38 (0.92 – 2.06), *0.12*, 262 | 1.64 (1.12 – 2.41), ***0.01***, 262 |
| *FCGR2C* (STP/ORF) | 2 copies (Ref) | -  312 | -  173 | -  173 |
|  | <2 copies | -0.06 (0.22), *0.80,* 36 | 1.43 (0.53 – 3.82), *0.48*, 23 | 1.92 (0.80 – 4.61), *0.15*, 23 |
|  | >2 copies | -0.23 (0.19), *0.25,* 50 | 1.71 (0.60 – 4.88), *0.31*, 22 | 3.02 (1.22 – 7.48), ***0.02***, 22 |
|  | Per copy of ORF allele | -0.09 (0.10), *0.36,* 398 | 1.55 (0.80 – 3.00), *0.19*, 220 | 1.93 (1.09 – 3.42), ***0.02***, 220 |
| *FCGR3B* (NA1/NA2 haplotype) | 2 copies (Ref) | -  334 | -  212 | -  212 |
|  | <2 copies | -0.12 (0.21), *0.57,* 40 | 1.22 (0.51 – 2.93), *0.65*, 27 | 1.52 (0.67 – 3.46), *0.32*, 27 |
|  | >2 copies | -0.02 (0.21), *0.92,* 41 | 2.31 (0.76 – 7.09), *0.14*, 22 | 3.20 (1.30 – 7.85), ***0.01***, 22 |
|  | Per copy of NA1 allele | 0.08 (0.09), *0.38,* 396 | 1.18 (0.81 – 1.72), *0.39*, 259 | 1.24 (0.86 – 1.78), *0.24*, 259 |

^1^Genes presented in chromosomal order on 1q23 centromere to telomere.

^2^Coefficient, standard error (SE) and p-value for the effect of the indicated genotype or copy number on outcome at 6-months compared with baseline genotype or copy number. Positive coefficients for clinical response outcomes indicate a worse outcome.

^3^ Odds ratio, 95% CI and p-value for the effect of the indicated copy number on outcome at 6 months.

^4^ *FCGR3A, FCGR2C* and *FCGR3B* are subject to copy number variation, analyses were performed according to biallelic genotype whereby the effect of heterozygosity and homozygosity for the rare allele were compared with homozygosity for the common allele.

^5^ All 5 individuals with a deletion achieved BILAG any response, so the OR was not estimated as the outcome was predicted perfectly

N: number

**Supplementary Table 7. Effect of *FCGR* genotype and copy number on the three-component DAS28 (3C-DAS28CRP) response to rituximab in RA**

| ***Gene^1^*** | **Genotype/ Copy Number** | **Rheumatoid Arthritis** |
| --- | --- | --- |
|  |  | **Effect on 3C-DAS28CRP at 6-month: coefficient (SE)^2^, *p-value*, N** |
| **Genotypic Analyses** | | |
| *FCGR2A* (Q27W) | Q  (Ref) | -  316 |
|  | QW | 0.00 (0.14), *0.99*, 92 |
|  | W | 0.98 (0.52), *0.06*, 5 |
|  | Additive model | 0.10 (0.12), *0.42*, 413 |
| *FCGR2A* (H131R) | R  (Ref) | -  102 |
|  | RH | -0.08 (0.14), *0.59*, 212 |
|  | H | 0.34 (0.16), ***0.03***, 99 |
|  | Additive model | 0.17 (0.08), *0.04*, 413 |
| *FCGR3A* (F158V) ^3^ | F  (Ref) | -  174 |
|  | FV | -0.08 (0.12), *0.53*, 192 |
|  | V | -0.21 (0.19), *0.27*, 47 |
|  | Additive model | -0.10 (0.09), *0.26*, 413 |
| *FCGR2C* (STP/ORF)^3^ | STP  (Ref) | -  287 |
|  | STPORF | 0.00 (0.14), *0.99*, 95 |
|  | ORF | -0.40 (0.32), *0.21*, 14 |
|  | Additive model | -0.08 (0.11), *0.45*, 396 |
| *FCGR3B*^3^  (NA1/NA2 haplotype) | NA2  (Ref) | -  171 |
|  | NA2NA1 | 0.26 (0.13), ***0.04***, 166 |
|  | NA1 | 0.12 (0.18), *0.52*, 57 |
|  | Additive model | 0.11 (0.08), *0.20*, 394 |
| *FCGR2B* (I123T) | I  (Ref) | -  325 |
|  | IT | 0.11 (0.14), *0.44*, 80 |
|  | T | -0.15 (0.48), *0.76*, 6 |
|  | Additive model | 0.06 (0.13), *0.61*, 411 |
| **Copy Number Analyses** | | |
| *FCGR3A* (F158V) | 2 copies (Ref) | -  373 |
|  | <2 copies | 0.47 (0.39), *0.23*, 9 |
|  | >2 copies | -0.31 (0.22), *0.16*, 31 |
|  | Per copy of V allele | -0.09 (0.08), *0.26*, 413 |
| *FCGR2C* (STP/ORF) | 2 copies (Ref) | -  311 |
|  | <2 copies | -0.03 (0.21), *0.88*, 35 |
|  | >2 copies | -0.26 (0.18), *0.15*, 50 |
|  | Per copy of ORF allele | -0.09 (0.09), *0.31*, 396 |
| *FCGR3B*  (NA1/NA2 haplotype) | 2 copies (Ref) | -  333 |
|  | <2 copies | -0.02 (0.20), *0.91*, 39 |
|  | >2 copies | -0.02 (0.19), *0.90*, 41 |
|  | Per copy of NA1 allele | 0.13 (0.08), *0.13*, 394 |

^1^Genes presented in chromosomal order on 1q23 centromere to telomere.

^2^Coefficient, standard error (SE) and p-value for the effect of the indicated genotype or copy number on outcome at 6-months compared with baseline genotype or copy number. Positive coefficients for clinical response outcomes indicate a worse outcome.

^3^ *FCGR3A, FCGR2C* and *FCGR3B* are subject to copy number variation, analyses were performed according to biallelic genotype whereby the effect of heterozygosity and homozygosity for the rare allele were compared with homozygosity for the common allele.

N: number

**Supplementary Table 8: Effect of *FCGR* genotype and copy number on clinical response to rituximab in Caucasians with SLE**

| ***Gene*** | **Genotypes/ Copy Number** | **BILAG Response (Major or Partial Clinical Response) at 6 months: OR (95% CI), *p-value*, N** | **BILAG Major Clinical Response at 6 months: OR (95% CI), *p-value*, N** |
| --- | --- | --- | --- |
| **Genotypic Analyses** | | | |
| *FCGR2A*  (Q27W) | Q  (Reference) | -  120 | -  120 |
|  | QW | 2.02 (0.85 – 4.82), *0.11*, 37 | 1.85 (0.86 - 3.96), *0.12*, 37 |
|  | W | 0.56 (0.08 – 4.11), *0.57*, 4 | 2.43 (0.33 – 18.01), *0.38*, 4 |
|  | Additive model | 1.39 (0.70 – 2.79), *0.35*, 161 | 1.74 (0.92 – 3.29), *0.09*, 161 |
| *FCGR2A*  (H131R) | R  (Reference) | -  44 | -  44 |
|  | RH | 0.98 (0.45 – 2.12), *0.96*, 84 | 1.20 (0.53 - 2.68), *0.67*, 84 |
|  | H | 1.38 (0.51 – 3.70), *0.52*, 33 | 2.22 (0.86 – 5.77), *0.10*, 33 |
|  | Additive model | 1.15 (0.71 – 1.86), *0.56*, 161 | 1.49 (0.92 – 2.41), *0.11*, 161 |
| *FCGR3A^4^* (F158V) | F  (Reference) | -  75 | -  75 |
|  | FV | 1.86 (0.92 - 3.74), *0.08*, 72 | 1.88 (0.93 - 3.79), *0.08*, 72 |
|  | V | 1.67 (0.48 – 5.81), *0.42*, 14 | 2.21 (0.68 – 7.19), *0.19*, 14 |
|  | Additive model | 1.53 (0.89 – 2.61), *0.12*, 161 | 1.62 (0.97 – 2.71), *0.07*, 161 |
| *FCGR2C^4^*  (STP/ORF) | STP  (Reference) | -  96 | -  96 |
|  | STPORF | 1.88 (0.74 - 4.81), *0.19*, 31 | 1.97 (0.86 - 4.48), *0.11*, 31 |
|  | ORF | 0.82 (0.13 – 5.17), *0.84*, 5 | 1.40 (0.22 – 8.80), *0.72*, 5 |
|  | Additive model | 1.34 (0.66 – 2.74), *0.42*, 132 | 1.57 (0.82 – 2.99), *0.17*, 132 |
| *FCGR3B^4^*  (NA1/NA2 haplotype) | NA2  (Reference) | -  74 | -  74 |
|  | NA2NA1 | 0.76 (0.38 – 1.52), *0.44*, 70 | 0.90 (0.45 – 1.80), *0.76*, 70 |
|  | NA1 | 1.47 (0.43 – 4.98), *0.54*, 17 | 1.07 (0.35 – 3.23), *0.91*, 17 |
|  | Additive model | 1.02 (0.62 – 1.67), *0.95*, 161 | 0.99 (0.60 – 1.62), *0.95*, 161 |
| *FCGR2B*  (I123T) | I  (Reference) | -  129 | -  129 |
|  | IT | 0.74 (0.33 - 1.66), *0.46,* 31 | 0.82 (0.35 - 1.93), *0.65*, 31 |
|  | T | See Note^1^, 1 | See Note^1^, 1 |
|  | Additive model | 0.84 (0.39 – 1.81), *0.65*, 161 | 1.02 (0.47 – 2.24), *0.96*, 161 |
| **Copy Number Analyses** | | | |
| *FCGR3A* (F158V) | 2 copies (reference) | -  151 | -  151 |
|  | <2 copies | See Note^2^, 5 | 0.48 (0.05 – 4.37), *0.51*, 5 |
|  | >2 copies | 0.33 (0.05 – 2.04), *0.23*, 5 | See Note^3^, 5 |
|  | Per copy of V allele | 1.53 (0.89 – 2.61), *0.12*, 161 | 1.62 (0.97 – 2.71), *0.07*, 161 |
| *FCGR2C* (STP/ORF) | 2 copies (reference) | -  108 | -  108 |
|  | <2 copies | 6.22 (0.77 – 48.99), *0.09*, 12 | 2.92 (0.87 – 9.85), *0.08*, 12 |
|  | >2 copies | 1.32 (0.32 – 5.39), *0.70*, 10 | 2.09 (0.57 – 7.68), *0.27*, 10 |
|  | Per copy of ORF allele | 1.34 (0.66 – 2.74), *0.42*, 132 | 1.75 (0.91 – 3.36), *0.09*, 132 |
| *FCGR3B* (NA1/NA2 haplotype) | 2 copies (reference) | -  135 | -  135 |
|  | <2 copies | 7.65 (0.97 – 60.22), *0.05*, 14 | 2.46 (0.81 – 7.48), *0.11*, 14 |
|  | >2 copies | 2.94 (0.62 – 13.97), *0.18*, 12 | 3.45 (1.03 – 11.52), ***0.04***, 12 |
|  | Per copy of NA1 allele | 1.04 (0.62 – 1.73), *0.88*, 161 | 1.03 (0.62 – 1.71), *0.91*, 161 |

^1^ The one individual with *FCGR2B*-232T homozygosity achieved British Isles Lupus Assessment Group (BILAG) any response, so the odds ratio (OR) was not estimated as the outcome was predicted perfectly

^2^ All 5 individuals with *FCGR3A* deletion showed BILAG response at 6 months, so the OR was not estimated as the outcome was predicted perfectly

^3^ All 5 individuals with *FCGR3A* duplication showed BILAG MCR at 6 months, so the OR was not estimated as the outcome was predicted perfectly

^4^ *FCGR3A, FCGR2C* and *FCGR3B* are subject to copy number variation, analyses were performed according to biallelic genotype whereby the effect of heterozygosity and homozygosity for the rare allele were compared with homozygosity for the common allele CI: confidence interval; N: number

**Supplementary Table 9: Baseline clinical characteristics and laboratory measures and association with complete B-cell depletion in the combined RA and SLE analyses**

| Characteristics | Sample Size | Complete B-cell depletion: Mean (SD) or number (%) positive | Incomplete B-cell depletion: Mean (SD) or number (%) positive | Complete B-cell depletion post-RTX: OR (95% CI), *p-value* |
| --- | --- | --- | --- | --- |
| Age at first RTX cycle (effect per 10 years) | 394 | 5.61 (1.44) | 5.26 (1.57) | 1.17 (1.02 – 1.34), ***0.02*** |
| Sex (Female) | 413 | 206 (87%) | 141 (80%) | 1.75 (1.03 – 2.98), ***0.04*** |
| Disease indication [i.e. RA as Reference] | 387 | 44 (52%) | 41 (48%) | 0.76 (0.47 – 1.23), *0.26* |
| Concomitant DMARDs^1^, including HCQ | 387 | 189 (86%) | 123 (74%) | 2.18 (1.31-3.64), ***0.003*** |
| Concomitant oral prednisolone^2^ | 85 | 33 (75%) | 30 (73.2%) | 1.10 (0.42 – 2.90), *0.85* |
| Oral prednisolone dose (mg/day)^2^ | 85 | 13.7 (12.6) | 13.6 (11.6) | 1.00 (0.97 – 1.04), *0.96* |
| IgM (g/L) | 397 | 1.4 (0.7) | 1.5 (1.1) | 0.84 (0.67 – 1.05), *0.13* |
| IgA (g/L) | 398 | 3.0 (1.3) | 3.4 (1.6) | 0.82 (0.71 – 0.95), ***0.01*** |
| IgG (g/L) | 398 | 12.1 (4.1) | 13.6 (5.2) | 0.93 (0.89 – 0.98), ***0.003*** |
| Total B-cell counts  (x 10^9^/L)^3^ | 395 | 124.3 (125) | 143.4 (145) | 1.00 (1.00 – 1.00), *0.16* |
| Naïve B-cell counts  (x 10^9^/L)^3^ | 393 | 93.3 (96) | 106.2 (123) | 1.00 (1.00 – 1.01), *0.25* |
| Memory B-cell counts (x 10^9^/L)^3^ | 393 | 28.6 (54) | 32.1 (34) | 1.00 (0.99 – 1.00), *0.47* |
| Plasmablast counts  (x 10^9^/L)^3^ | 395 | 2.3 (3) | 5.8 (9) | 0.84 (0.78 – 0.89), ***<0.001*** |

^1^ Concomitant disease modifying anti-rheumatic drugs (DMARDs)

^2^ Data available for systemic lupus erythematosus (SLE) Leeds cohort only

^3^ Count x 10^9^ cells/L for each subset multiplied by 1000 prior to analysis

**Supplementary Table 10. Effect of *FCGR* genotype and copy number on complete B-cell depletion following rituximab in the combined RA and SLE analyses**

| ***Gene ^1^*** | **Genotype/**  **Copy Number** | **Complete B-cell Depletion: N (%)** | **Incomplete B-cell Depletion: N (%)** | **Unadjusted Complete B-cell Depletion post-RTX:**  **OR (95% CI), *p-value*^2^** | **Adjusted Complete B-cell Depletion post-RTX^3^:**  **OR (95% CI), *p-value*^2^** |
| --- | --- | --- | --- | --- | --- |
| **Genotypic Analyses** | | | | | |
| *FCGR2A*  (Q27W) | Q  (Reference) | 173 (73.3) | 138 (78.0) | - | - |
|  | QW | 59 (25.0) | 34 (19.2) | 1.38 (0.86 – 2.23), *0.18* | 1.22 (0.71 – 2.11), *0.46* |
|  | W | 4 (1.7) | 5 (2.8) | 0.64 (0.17 – 2.42), *0.51* | 1.28 (0.27 – 5.99), *0.76* |
|  | Additive model | - | - | 1.16 (0.78 – 1.73), *0.47* | 1.19 (0.75 – 1.89), *0.45* |
| *FCGR2A*  (H131R) | R  (Reference) | 63 (26.7) | 47 (26.6) | - | - |
|  | RH | 114 (48.3) | 94 (53.1) | 0.90 (0.57 – 1.44), *0.67* | 0.74 (0.43 – 1.27), *0.27* |
|  | H | 59 (25.0) | 36 (20.3) | 1.22 (0.70 – 2.14), *0.48* | 1.29 (0.67 – 2.48), *0.44* |
|  | Additive model | - | - | 1.10 (0.83 – 1.45), *0.52* | 1.12 (0.81 – 1.54), *0.48* |
| *FCGR3A*^4^ (F158V) | F  (Reference) | 95 (40.3) | 86 (48.6) | - | - |
|  | FV | 114 (48.3) | 80 (45.2) | 1.29 (0.86 – 1.94), *0.22* | 1.13 (0.70 - 1.82), *0.61* |
|  | V | 27 (11.4) | 11 (6.2) | 2.22 (1.04 – 4.75), ***0.04*** | 3.03 (1.23 – 7.42), ***0.02*** |
|  | Additive model | - | - | 1.40 (1.03 – 1.91), ***0.03*** | 1.44 (1.01 – 2.06), ***0.04*** |
| *FCGR2C*^4^  (STP/ORF) | STP  (Reference) | 156 (70.0) | 126 (75.0) | - | - |
|  | STPORF | 59 (26.5) | 36 (21.4) | 1.32 (0.82 – 2.13), *0.25* | 1.28 (0.75 - 2.21), *0.36* |
|  | ORF | 8 (3.6) | 6 (3.6) | 1.08 (0.36 – 3.18), *0.89* | 1.51 (0.41 – 5.58), *0.54* |
|  | Additive model | - | - | 1.20 (0.82 – 1.75), *0.36* | 1.26 (0.81 – 1.96), *0.30* |
| *FCGR3B*^4^  (NA1/NA2 haplotype) | NA2  (Reference) | 98 (44.0) | 65 (40.4) | - | - |
|  | NA2NA1 | 98 (44.0) | 75 (46.6) | 0.87 (0.56 – 1.34), *0.52* | 1.04 (0.62 - 1.74), *0.88* |
|  | NA1 | 27 (12.1) | 21 (13.0) | 0.85 (0.44 – 1.63), *0.63* | 0.74 (0.36 – 1.55), *0.43* |
|  | Additive model | - | - | 0.91 (0.67 – 1.22), *0.52* | 0.90 (0.64 – 1.28), *0.57* |
| *FCGR2B*  (I123T) | I  (Reference) | 184 (78.3) | 137 (78.3) | - | - |
|  | IT | 48 (20.4) | 36 (20.6) | 0.99 (0.61 – 1.61), *0.98* | 0.87 (0.49 - 1.54), *0.63* |
|  | T | 3 (1.3) | 2 (1.1) | 1.12 (0.18 – 6.78), *0.90* | 0.43 (0.06 – 3.21), *0.41* |
|  | Additive model | - | - | 1.01 (0.65 – 1.56), *0.98* | 0.82 (0.49 – 1.36), *0.44* |
| **Copy Number Analyses** | | | | | |
| *FCGR3A* (F158V) | 2 copies (reference) | 208 (88.1) | 165 (93.2) | - | - |
|  | <2 copies | 5 (2.1) | 1 (0.6) | 3.97 (0.46 – 34.28), *0.21* | 3.12 (0.33 – 29.57), *0.32* |
|  | >2 copies | 23 (9.8) | 11 (6.2) | 1.66 (0.79 – 3.50), *0.18* | 2.35 (0.99 – 5.57), ***0.05*** |
|  | Per copy of V allele | 141 (59.7) | 91 (51.4) | 1.41 (1.05 – 1.90), ***0.02*** | 1.49 (1.06 – 2.08), ***0.02*** |
| *FCGR2C* (STP/ORF) | 2 copies (reference) | 172 (77.1) | 135 (80.4) | - | - |
|  | <2 copies | 22 (9.9) | 12 (7.1) | 1.44 (0.69 – 3.01), *0.33* | 1.23 (0.52 – 2.88), *0.64* |
|  | >2 copies | 29 (13.0) | 21 (12.5) | 1.08 (0.59 – 1.99), *0.79* | 1.28 (0.65 – 2.54), *0.48* |
|  | Per copy of ORF allele | 67 (30.0) | 42 (25.0) | 1.23 (0.87 – 1.75), *0.24* | 1.35 (0.90 – 2.02), *0.15* |
| *FCGR3B* (NA1/NA2 haplotype) | 2 copies (reference) | 191 (80.9) | 146 (82.5) | - | - |
|  | <2 copies | 22 (9.3) | 14 (7.9) | 1.20 (0.59 – 2.43), *0.61* | 1.13 (0.51 – 2.54), *0.76* |
|  | >2 copies | 23 (9.8) | 17 (9.6) | 1.03 (0.53 – 2.01), *0.92* | 1.14 (0.54 – 2.40), *0.73* |
|  | Per copy of NA1 allele | 131 (57.2) | 104 (61.5) | 0.85 (0.64 – 1.14), *0.28* | 0.91 (0.65 – 1.27), *0.56* |

^1^Genes presented in chromosomal order on 1q23 centromere to telomere.

^2^Odds ratio (OR), 95% confidence intervals (CI) and p-value for the effect of the indicated genotype or copy number on complete B-cell depletion at 2 weeks, compared with reference genotype. All tests were performed using logistic regression.

^3^Analyses adjusted for age, concomitant disease-modifying anti-rheumatic drug, including hydroxychloroquine, and baseline plasmablast count.

*^4^FCGR3A, FCGR2C* and *FCGR3B* are subject to copy number variation, analyses were performed according to biallelic genotype whereby the effect of heterozygosity and homozygosity for the rare allele were compared with homozygosity for the common allele.

N: number
