# Supplementary material for: Comprehensive genetic and functional analyses of Fc gamma receptors influence on response to rituximab therapy for autoimmunity
Source: eBioMedicine. 2022 Nov 11;86:104343. doi: 10.1016/j.ebiom.2022.104343 (PMC9663864; doi:10.1016/j.ebiom.2022.104343)
Supplement: Supplementary Tables S1–S10 [file mmc2.docx]

**Supplementary Table 1. Oligonucleotide primer sequences used to sequence *FCGR2* genes and a *FCGR2C* QSV assay**

| Gene | Forward | Reverse | Amplicon length (bp) |
| --- | --- | --- | --- |
| *FCGR2A* | dGTGAGCATTTTAGTACCAGTTGCTTTGAC | dCCTTTAACAATTCCCCTCTTTTTGTCATCCACTC | 21,264 |
| *FCGR2B* | dCTCCACAGGTTACTCGTTTCTACCTTATCTTAC | dCCCAGAAAGAATCACTTTTAATGTGCTGG | 16,660 |
| *FCGR2C* | dCTCCACAGGTTACTCGTTTCTACCTTATCTTAC | dCCTTTAACAATTCCCCTCTTTTTGTCATCCACTC | 20,371 |
| *FCGR2A, B & C* | dAGTTCAGCTGGGAGCCAGGGA | dGCCTCAGTCTTACAGCCCCTA (QSV seq primer) | 279 |

**Supplementary Table 2.** ***FCGR* genotype frequencies and Hardy Weinberg equilibrium in the RA and SLE cohorts**

|  |  | **RA (n=611)** | **%** | **HWP^1^** | **SLE (n=594)** | **%** | **HWP^1^** |
| --- | --- | --- | --- | --- | --- | --- | --- |
| ***FCGR2A* Q27W** | QQ | 465 | 76.1 | 0.99 | 458 | 77.1 | 0.33 |
|  | QW | 136 | 22.3 |  | 130 | 21.9 |  |
|  | WW | 10 | 1.6 |  | 6 | 1.0 |  |
|  | Fail/unclear | 0 | 0.0 |  | 0 | 0.0 |  |
| ***FCGR2A* H131R** | HH | 152 | 24.9 | 0.84 | 140 | 23.6 | 0.49 |
|  | HR | 303 | 49.6 |  | 288 | 48.5 |  |
|  | RR | 156 | 25.5 |  | 166 | 27.9 |  |
|  | Fail/unclear | 0 | 0.0 |  | 0 | 0.0 |  |
| ***FCGR3A* F158V** | FF | 243 | 39.8 | 0.75 | 232 | 39.1 | 0.73 |
|  | FV | 247 | 40.4 |  | 245 | 41.2 |  |
|  | VV | 59 | 9.7 |  | 69 | 11.6 |  |
|  | F | 8 | 1.3 |  | 6 | 1.0 |  |
|  | V | 4 | 0.7 |  | 3 | 0.5 |  |
|  | FFF | 4 | 0.7 |  | 13 | 2.2 |  |
|  | FFV | 26 | 4.3 |  | 19 | 3.2 |  |
|  | FVV | 14 | 2.3 |  | 3 | 0.5 |  |
|  | VVV | 5 | 0.8 |  | 1 | 0.2 |  |
|  | FFFV | 0 | 0.0 |  | 1 | 0.2 |  |
|  | FFVV | 1 | 0.2 |  | 0 | 0.0 |  |
|  | Fail/Unclear | 0 | 0.0 |  | 2 | 0.3 |  |
| ***FCGR2C* X57Q** | ORF/ORF | 11 | 1.8 | 0.62 | 9 | 1.5 | 0.70 |
| **(classical STP/ORF)** | STP/ORF | 111 | 18.2 |  | 99 | 16.7 |  |
|  | STP/STP | 335 | 54.8 |  | 317 | 53.4 |  |
|  | del | 0 | 0.0 |  | 2 | 0.3 |  |
|  | ORF | 5 | 0.8 |  | 8 | 1.3 |  |
|  | STP | 42 | 6.9 |  | 60 | 10.1 |  |
|  | ORF/ORF/ORF | 3 | 0.5 |  | 3 | 0.5 |  |
|  | STP/ORF/ORF | 17 | 2.8 |  | 14 | 2.4 |  |
|  | STP/STP/ORF | 10 | 1.6 |  | 17 | 2.9 |  |
|  | STP/STP/STP | 35 | 5.7 |  | 55 | 9.3 |  |
|  | ORF/ORF/ORF/ORF | 1 | 0.2 |  | 0 | 0.0 |  |
|  | STP/ORF/ORF/ORF | 0 | 0.0 |  | 1 | 0.2 |  |
|  | STP/STP/ORF/ORF | 0 | 0.0 |  | 2 | 0.3 |  |
|  | STP/STP/STP/ORF | 2 | 0.3 |  | 2 | 0.3 |  |
|  | STP/STP/STP/STP | 6 | 1.0 |  | 4 | 0.7 |  |
|  | Fail/Unclear | 33 | 5.4 |  | 1 | 0.2 |  |
| ***FCGR3B* NA1/NA2/SH** | NA1/NA1 | 63 | 10.3 | 0.16 | 72 | 12.1 | 0.05 |
|  | NA1/NA2 | 201 | 32.9 |  | 184 | 31.0 |  |
|  | NA2/NA2 | 213 | 34.9 |  | 176 | 29.6 |  |
|  | NA1/SH | 9 | 1.5 |  | 9 | 1.5 |  |
|  | NA2/SH | 3 | 0.5 |  | 7 | 1.2 |  |
|  | SH/SH | 1 | 0.2 |  | 2 | 0.3 |  |
|  | del | 0 | 0.0 |  | 2 | 0.3 |  |
|  | NA1 | 17 | 2.8 |  | 29 | 4.9 |  |
|  | NA2 | 37 | 6.1 |  | 33 | 5.6 |  |
|  | SH | 0 | 0.0 |  | 4 | 0.7 |  |
|  | NA1/NA1/NA1 | 0 | 0.0 |  | 1 | 0.2 |  |
|  | NA1/NA1/NA2 | 14 | 2.3 |  | 21 | 3.5 |  |
|  | NA1/NA1/SH | 0 | 0.0 |  | 4 | 0.7 |  |
|  | NA1/NA2/NA2 | 18 | 2.9 |  | 22 | 3.7 |  |
|  | NA1/NA2/SH | 0 | 0.0 |  | 10 | 1.7 |  |
|  | NA1/SH/SH | 5 | 0.8 |  | 5 | 0.8 |  |
|  | NA2/NA2/NA2 | 1 | 0.2 |  | 4 | 0.7 |  |
|  | NA2/NA2/SH | 0 | 0.0 |  | 2 | 0.3 |  |
|  | NA1/NA1/NA2/NA2 | 0 | 0.0 |  | 1 | 0.2 |  |
|  | NA1/NA1/NA2/SH | 0 | 0.0 |  | 2 | 0.3 |  |
|  | NA1/NA2/NA2/NA2 | 0 | 0.0 |  | 1 | 0.2 |  |
|  | NA2/NA2/NA2/SH | 0 | 0.0 |  | 2 | 0.3 |  |
|  | Fail/Unclear | 29 | 4.7 |  | 1 | 0.2 |  |
| ***FCGR2B* I232T** | II | 475 | 77.7 | 0.98 | 442 | 74.4 | 0.50 |
|  | IT | 124 | 20.3 |  | 143 | 24.1 |  |
|  | TT | 8 | 1.3 |  | 9 | 1.5 |  |
|  | Fail/Unclear | 4 | 0.7 |  | 0 | 0.0 |  |

^1^ Hardy Weinberg equilibrium P value, using diploid genotypes only (shaded rows).

^2^ *FCGR2C* quantitative genotypes were generated using the gene copy number derived from our *FCGR2C* QSV assay as an independent measure.

**Supplementary Table 3. *FCGR3A*, *FCGR2C* and *FCGR3B* gene copy number frequencies in RA and SLE cohorts**

|  |  | **RA** | | **SLE** | | |
| --- | --- | --- | --- | --- | --- | --- |
|  | **Copy number** | **n** | **%** | **n** | **%** |  |
| ***FCGR3A*** | 1 | 12 | 2.0 | 9 | 1.5 | |
|  | 2 | 548 | 89.7 | 547 | 92.2 | |
|  | 3 | 50 | 8.2 | 36 | 6.1 | |
|  | 4 | 1 | 0.2 | 1 | 0.2 | |
| ***FCGR2C*** | 0 | 0 | 0.0 | 2 | 0.3 | |
|  | 1 | 47 | 8.1 | 68 | 11.4 | |
|  | 2 | 457 | 79.1 | 425 | 71.5 | |
|  | 3 | 65 | 11.2 | 90 | 15.2 | |
|  | 4 | 9 | 1.6 | 9 | 1.5 | |
| ***FCGR3B*** | 0 | 0 | 0.0 | 2 | 0.3 | |
|  | 1 | 54 | 8.8 | 66 | 11.1 | |
|  | 2 | 497 | 81.3 | 453 | 76.3 | |
|  | 3 | 53 | 8.7 | 67 | 11.3 | |
|  | 4 | 7 | 1.1 | 6 | 1.0 | |

^1^ *FCGR2C* gene copy number was determined using our QSV assay to complement the limited number of *FCGR2C* specific probes on the B2 version of the P110 and P111 MLPA panels.

**Supplementary Table 4. Copy number region loss and gain frequencies for RA and SLE cohorts**

| **CNR event** | **MATURA** | **BILAG-BR** |
| --- | --- | --- |
| CNR1 gain | 45 (3.7%) | 77 (6.5%) |
| CNR1 loss | 43 (3.5%) | 64 (5.4%) |
| CNR2 gain | 23 (1.9%) | 32 (2.7%) |
| CNR2 loss | 4 (0.3%) | 6 (0.5%) |
| CNR1&2 gain | 4 (0.3%) | 4 (0.3%) |
| CNR1&2 loss | 0 | 0 |

**Supplementary Table 5. Pairwise linkage disequilibrium (r^2^) between biallelic diploid SNP- based markers in the *FCGR* locus in 127 confirmed British Caucasian SLE participants restricted to individuals with two copies of *FCGR3A*, *FCGR2C* and *FCGR3B***

|  | ***FCGR2A* Q27W** | ***FCGR2A* H131R** | ***FCGR3A* F158V** | ***FCGR2C* cSTP/ORF** | ***FCGR3B* NA1/NA2** | ***FCGR2B* I232T** |
| --- | --- | --- | --- | --- | --- | --- |
| ***FCGR2A* Q27W** |  | 0.18 | 0.19 | 0.61 | 0.01 | 0.00 |
| ***FCGR2A* H131R** |  |  | 0.18 | 0.08 | 0.02 | 0.00 |
| ***FCGR3A* F158V** |  |  |  | 0.29 | 0.14 | 0.00 |
| ***FCGR2C* cSTP/ORF** |  |  |  |  | 0.03 | 0.01 |
| ***FCGR3B* NA1/NA2** |  |  |  |  |  | 0.03 |

**Supplementary Table 6. Effect of *FCGR* genotype and copy number on clinical response to rituximab in RA and SLE**

| ***Gene^1^*** | **Genotypes/**  **Copy Number** | **Rheumatoid Arthritis** | **Systemic Lupus Erythematosus** | |
| --- | --- | --- | --- | --- |
|  |  | **Effect on 2C-DAS28CRP at 6-month: coefficient (SE)^2^, *p-value*, N** | **BILAG Response (Major or Partial) at 6 months: OR (95% CI)^3^, *p-value*, N** | **BILAG Major Clinical Response at 6 months: OR (95% CI)^3^, *p-value*, N** |
| **Genotypic Analyses** | | | | |
| *FCGR2A*  (Q27W) | Q  (Ref) | -  318 | -  208 | -  208 |
|  | QW | -0.01 (0.15), *0.97*, 92 | 2.01 (0.95 - 4.27), *0.07*, 48 | 1.67 (0.88 - 3.18), *0.12*, 48 |
|  | W | 0.51 (0.57), *0.38*, 5 | 0.53 (0.07 - 3.84), *0.53*, 4 | 2.15 (0.30 - 15.61), *0.45*, 4 |
|  | Additive model | 0.05 (0.14), 0.74, 415 | 1.47 (0.79 – 2.75), *0.23*, 260 | 1.61 (0.93 – 2.81), *0.09*, 260 |
| *FCGR2A*  (H131R) | R  (Ref) | -  103 | -  70 | -  70 |
|  | RH | -0.18 (0.15), *0.25*, 212 | 0.74 (0.40 - 1.38), *0.35*, 134 | 1.14 (0.61 - 2.15), *0.68*, 134 |
|  | H | 0.21 (0.18), *0.24*, 100 | 1.23 (0.56 - 2.68), *0.60*, 58 | 2.33 (1.12 - 4.85), ***0.02***, 58 |
|  | Additive model | 0.10 (0.09), 0.25, 415 | 1.08 (0.74 – 1.56), *0.69*, 262 | 1.53 (1.06 – 2.22), ***0.03***, 262 |
| *FCGR3A^4^* (F158V) | F  (Ref) | -  175 | -  126 | -  126 |
|  | FV | -0.29 (0.13), ***0.03***, 193 | 1.93 (1.10 - 3.39), ***0.02***, 109 | 1.76 (1.02 - 3.06), ***0.04***, 109 |
|  | V | -0.28 (0.21), *0.17*, 47 | 1.27 (0.53 - 3.06), *0.59*, 27 | 2.51 (1.07 - 5.89), ***0.03***, 27 |
|  | Additive model | -0.19 (0.09). **0.04**, 415 | 1.38 (0.92 – 2.06), *0.12*, 262 | 1.64 (1.12 – 2.41), ***0.01***, 262 |
| *FCGR2C*^4^  (STP/ORF) | STP  (Ref) | -  289 | -  173 | -  173 |
|  | STPORF | -0.02 (0.15), *0.89*, 95 | 2.20 (0.96 - 5.05), *0.06*, 42 | 2.24 (1.13 - 4.43), ***0.02***, 42 |
|  | ORF | -0.27 (0.35), *0.45*, 14 | 0.78 (0.13 - 4.78), *0.79*, 5 | 1.36 (0.22 - 8.35), *0.74*, 5 |
|  | Additive model | -0.07 (0.12), 0.57, 398 | 1.55 (0.80 – 3.00), *0.19*, 220 | 1.77 (1.01 – 3.13), ***0.05***, 220 |
| *FCGR3B*^4^  (NA1/NA2 haplotype) | NA2  (Ref) | -  172 | -  106 | -  106 |
|  | NA2NA1 | 0.19 (0.14), *0.19*, 166 | 1.03 (0.58 - 1.81), *0.93*, 111 | 1.01 (0.58 - 1.79), *0.96*, 111 |
|  | NA1 | -0.02 (0.19), *0.91*, 58 | 1.23 (0.56 - 2.69), *0.60*, 42 | 1.52 (0.73 - 3.17), *0.26*, 42 |
|  | Additive model | 0.04 (0.09), 0.69, 396 | 1.09 (0.76 – 1.58), *0.64*, 259 | 1.19 (0.83 – 1.70), *0.34*, 259 |
| *FCGR2B*  (I123T) | I  (Ref) | -  327 | -  197 | -  197 |
|  | IT | 0.05 (0.16), *0.75*, 80 | 0.68 (0.37 - 1.23), *0.20,* 61 | 0.84 (0.45 - 1.55), *0.5*8, 61 |
|  | T | -0.37 (0.53), *0.49*, 6 | 1.31 (0.13 - 12.89), *0.82*, 4 | 1.86 (0.26 - 13.46), *0.54*, 4 |
|  | Additive model | -0.01 (0.14), 0.95, 413 | 0.76 (0.45 – 1.30), *0.32*, 262 | 0.95 (0.55 – 1.63), *0.85*, 262 |
| **Copy Number Analyses** | | | | |
| *FCGR3A* (F158V) | 2 copies (Ref) | -  375 | -  248 | -  248 |
|  | <2 copies | 0.83 (0.43), ***0.05****,* 9 | See Note^5^, 5 | 0.46 (0.05 – 4.20), *0.49*, 5 |
|  | >2 copies | -0.52 (0.24), ***0.03****,* 31 | 0.61 (0.16 – 2.32), *0.47*, 9 | 0.53 (0.11 – 2.60), *0.43*, 9 |
|  | Per copy of V allele | -0.20 (0.09), ***0.02****,* 415 | 1.38 (0.92 – 2.06), *0.12*, 262 | 1.64 (1.12 – 2.41), ***0.01***, 262 |
| *FCGR2C* (STP/ORF) | 2 copies (Ref) | -  312 | -  173 | -  173 |
|  | <2 copies | -0.06 (0.22), *0.80,* 36 | 1.43 (0.53 – 3.82), *0.48*, 23 | 1.92 (0.80 – 4.61), *0.15*, 23 |
|  | >2 copies | -0.23 (0.19), *0.25,* 50 | 1.71 (0.60 – 4.88), *0.31*, 22 | 3.02 (1.22 – 7.48), ***0.02***, 22 |
|  | Per copy of ORF allele | -0.09 (0.10), *0.36,* 398 | 1.55 (0.80 – 3.00), *0.19*, 220 | 1.93 (1.09 – 3.42), ***0.02***, 220 |
| *FCGR3B* (NA1/NA2 haplotype) | 2 copies (Ref) | -  334 | -  212 | -  212 |
|  | <2 copies | -0.12 (0.21), *0.57,* 40 | 1.22 (0.51 – 2.93), *0.65*, 27 | 1.52 (0.67 – 3.46), *0.32*, 27 |
|  | >2 copies | -0.02 (0.21), *0.92,* 41 | 2.31 (0.76 – 7.09), *0.14*, 22 | 3.20 (1.30 – 7.85), ***0.01***, 22 |
|  | Per copy of NA1 allele | 0.08 (0.09), *0.38,* 396 | 1.18 (0.81 – 1.72), *0.39*, 259 | 1.24 (0.86 – 1.78), *0.24*, 259 |

^1^Genes presented in chromosomal order on 1q23 centromere to telomere.

^2^Coefficient, standard error (SE) and p-value for the effect of the indicated genotype or copy number on outcome at 6-months compared with baseline genotype or copy number. Positive coefficients for clinical response outcomes indicate a worse outcome.

^3^ Odds ratio, 95% CI and p-value for the effect of the indicated copy number on outcome at 6 months.

^4^ *FCGR3A, FCGR2C* and *FCGR3B* are subject to copy number variation, analyses were performed according to biallelic genotype whereby the effect of heterozygosity and homozygosity for the rare allele were compared with homozygosity for the common allele.

^5^ All 5 individuals with a deletion achieved BILAG any response, so the OR was not estimated as the outcome was predicted perfectly

N: number

**Supplementary Table 7. Effect of *FCGR* genotype and copy number on the three-component DAS28 (3C-DAS28CRP) response to rituximab in RA**

| ***Gene^1^*** | **Genotype/ Copy Number** | **Rheumatoid Arthritis** |
| --- | --- | --- |
|  |  | **Effect on 3C-DAS28CRP at 6-month: coefficient (SE)^2^, *p-value*, N** |
| **Genotypic Analyses** | | |
| *FCGR2A* (Q27W) | Q  (Ref) | -  316 |
|  | QW | 0.00 (0.14), *0.99*, 92 |
|  | W | 0.98 (0.52), *0.06*, 5 |
|  | Additive model | 0.10 (0.12), *0.42*, 413 |
| *FCGR2A* (H131R) | R  (Ref) | -  102 |
|  | RH | -0.08 (0.14), *0.59*, 212 |
|  | H | 0.34 (0.16), ***0.03***, 99 |
|  | Additive model | 0.17 (0.08), *0.04*, 413 |
| *FCGR3A* (F158V) ^3^ | F  (Ref) | -  174 |
|  | FV | -0.08 (0.12), *0.53*, 192 |
|  | V | -0.21 (0.19), *0.27*, 47 |
|  | Additive model | -0.10 (0.09), *0.26*, 413 |
| *FCGR2C* (STP/ORF)^3^ | STP  (Ref) | -  287 |
|  | STPORF | 0.00 (0.14), *0.99*, 95 |
|  | ORF | -0.40 (0.32), *0.21*, 14 |
|  | Additive model | -0.08 (0.11), *0.45*, 396 |
| *FCGR3B*^3^  (NA1/NA2 haplotype) | NA2  (Ref) | -  171 |
|  | NA2NA1 | 0.26 (0.13), ***0.04***, 166 |
|  | NA1 | 0.12 (0.18), *0.52*, 57 |
|  | Additive model | 0.11 (0.08), *0.20*, 394 |
| *FCGR2B* (I123T) | I  (Ref) | -  325 |
|  | IT | 0.11 (0.14), *0.44*, 80 |
|  | T | -0.15 (0.48), *0.76*, 6 |
|  | Additive model | 0.06 (0.13), *0.61*, 411 |
| **Copy Number Analyses** | | |
| *FCGR3A* (F158V) | 2 copies (Ref) | -  373 |
|  | <2 copies | 0.47 (0.39), *0.23*, 9 |
|  | >2 copies | -0.31 (0.22), *0.16*, 31 |
|  | Per copy of V allele | -0.09 (0.08), *0.26*, 413 |
| *FCGR2C* (STP/ORF) | 2 copies (Ref) | -  311 |
|  | <2 copies | -0.03 (0.21), *0.88*, 35 |
|  | >2 copies | -0.26 (0.18), *0.15*, 50 |
|  | Per copy of ORF allele | -0.09 (0.09), *0.31*, 396 |
| *FCGR3B*  (NA1/NA2 haplotype) | 2 copies (Ref) | -  333 |
|  | <2 copies | -0.02 (0.20), *0.91*, 39 |
|  | >2 copies | -0.02 (0.19), *0.90*, 41 |
|  | Per copy of NA1 allele | 0.13 (0.08), *0.13*, 394 |

^1^Genes presented in chromosomal order on 1q23 centromere to telomere.

^2^Coefficient, standard error (SE) and p-value for the effect of the indicated genotype or copy number on outcome at 6-months compared with baseline genotype or copy number. Positive coefficients for clinical response outcomes indicate a worse outcome.

^3^ *FCGR3A, FCGR2C* and *FCGR3B* are subject to copy number variation, analyses were performed according to biallelic genotype whereby the effect of heterozygosity and homozygosity for the rare allele were compared with homozygosity for the common allele.

N: number

**Supplementary Table 8: Effect of *FCGR* genotype and copy number on clinical response to rituximab in Caucasians with SLE**

| ***Gene*** | **Genotypes/ Copy Number** | **BILAG Response (Major or Partial Clinical Response) at 6 months: OR (95% CI), *p-value*, N** | **BILAG Major Clinical Response at 6 months: OR (95% CI), *p-value*, N** |
| --- | --- | --- | --- |
| **Genotypic Analyses** | | | |
| *FCGR2A*  (Q27W) | Q  (Reference) | -  120 | -  120 |
|  | QW | 2.02 (0.85 – 4.82), *0.11*, 37 | 1.85 (0.86 - 3.96), *0.12*, 37 |
|  | W | 0.56 (0.08 – 4.11), *0.57*, 4 | 2.43 (0.33 – 18.01), *0.38*, 4 |
|  | Additive model | 1.39 (0.70 – 2.79), *0.35*, 161 | 1.74 (0.92 – 3.29), *0.09*, 161 |
| *FCGR2A*  (H131R) | R  (Reference) | -  44 | -  44 |
|  | RH | 0.98 (0.45 – 2.12), *0.96*, 84 | 1.20 (0.53 - 2.68), *0.67*, 84 |
|  | H | 1.38 (0.51 – 3.70), *0.52*, 33 | 2.22 (0.86 – 5.77), *0.10*, 33 |
|  | Additive model | 1.15 (0.71 – 1.86), *0.56*, 161 | 1.49 (0.92 – 2.41), *0.11*, 161 |
| *FCGR3A^4^* (F158V) | F  (Reference) | -  75 | -  75 |
|  | FV | 1.86 (0.92 - 3.74), *0.08*, 72 | 1.88 (0.93 - 3.79), *0.08*, 72 |
|  | V | 1.67 (0.48 – 5.81), *0.42*, 14 | 2.21 (0.68 – 7.19), *0.19*, 14 |
|  | Additive model | 1.53 (0.89 – 2.61), *0.12*, 161 | 1.62 (0.97 – 2.71), *0.07*, 161 |
| *FCGR2C^4^*  (STP/ORF) | STP  (Reference) | -  96 | -  96 |
|  | STPORF | 1.88 (0.74 - 4.81), *0.19*, 31 | 1.97 (0.86 - 4.48), *0.11*, 31 |
|  | ORF | 0.82 (0.13 – 5.17), *0.84*, 5 | 1.40 (0.22 – 8.80), *0.72*, 5 |
|  | Additive model | 1.34 (0.66 – 2.74), *0.42*, 132 | 1.57 (0.82 – 2.99), *0.17*, 132 |
| *FCGR3B^4^*  (NA1/NA2 haplotype) | NA2  (Reference) | -  74 | -  74 |
|  | NA2NA1 | 0.76 (0.38 – 1.52), *0.44*, 70 | 0.90 (0.45 – 1.80), *0.76*, 70 |
|  | NA1 | 1.47 (0.43 – 4.98), *0.54*, 17 | 1.07 (0.35 – 3.23), *0.91*, 17 |
|  | Additive model | 1.02 (0.62 – 1.67), *0.95*, 161 | 0.99 (0.60 – 1.62), *0.95*, 161 |
| *FCGR2B*  (I123T) | I  (Reference) | -  129 | -  129 |
|  | IT | 0.74 (0.33 - 1.66), *0.46,* 31 | 0.82 (0.35 - 1.93), *0.65*, 31 |
|  | T | See Note^1^, 1 | See Note^1^, 1 |
|  | Additive model | 0.84 (0.39 – 1.81), *0.65*, 161 | 1.02 (0.47 – 2.24), *0.96*, 161 |
| **Copy Number Analyses** | | | |
| *FCGR3A* (F158V) | 2 copies (reference) | -  151 | -  151 |
|  | <2 copies | See Note^2^, 5 | 0.48 (0.05 – 4.37), *0.51*, 5 |
|  | >2 copies | 0.33 (0.05 – 2.04), *0.23*, 5 | See Note^3^, 5 |
|  | Per copy of V allele | 1.53 (0.89 – 2.61), *0.12*, 161 | 1.62 (0.97 – 2.71), *0.07*, 161 |
| *FCGR2C* (STP/ORF) | 2 copies (reference) | -  108 | -  108 |
|  | <2 copies | 6.22 (0.77 – 48.99), *0.09*, 12 | 2.92 (0.87 – 9.85), *0.08*, 12 |
|  | >2 copies | 1.32 (0.32 – 5.39), *0.70*, 10 | 2.09 (0.57 – 7.68), *0.27*, 10 |
|  | Per copy of ORF allele | 1.34 (0.66 – 2.74), *0.42*, 132 | 1.75 (0.91 – 3.36), *0.09*, 132 |
| *FCGR3B* (NA1/NA2 haplotype) | 2 copies (reference) | -  135 | -  135 |
|  | <2 copies | 7.65 (0.97 – 60.22), *0.05*, 14 | 2.46 (0.81 – 7.48), *0.11*, 14 |
|  | >2 copies | 2.94 (0.62 – 13.97), *0.18*, 12 | 3.45 (1.03 – 11.52), ***0.04***, 12 |
|  | Per copy of NA1 allele | 1.04 (0.62 – 1.73), *0.88*, 161 | 1.03 (0.62 – 1.71), *0.91*, 161 |

^1^ The one individual with *FCGR2B*-232T homozygosity achieved British Isles Lupus Assessment Group (BILAG) any response, so the odds ratio (OR) was not estimated as the outcome was predicted perfectly

^2^ All 5 individuals with *FCGR3A* deletion showed BILAG response at 6 months, so the OR was not estimated as the outcome was predicted perfectly

^3^ All 5 individuals with *FCGR3A* duplication showed BILAG MCR at 6 months, so the OR was not estimated as the outcome was predicted perfectly

^4^ *FCGR3A, FCGR2C* and *FCGR3B* are subject to copy number variation, analyses were performed according to biallelic genotype whereby the effect of heterozygosity and homozygosity for the rare allele were compared with homozygosity for the common allele CI: confidence interval; N: number

**Supplementary Table 9: Baseline clinical characteristics and laboratory measures and association with complete B-cell depletion in the combined RA and SLE analyses**

| Characteristics | Sample Size | Complete B-cell depletion: Mean (SD) or number (%) positive | Incomplete B-cell depletion: Mean (SD) or number (%) positive | Complete B-cell depletion post-RTX: OR (95% CI), *p-value* |
| --- | --- | --- | --- | --- |
| Age at first RTX cycle (effect per 10 years) | 394 | 5.61 (1.44) | 5.26 (1.57) | 1.17 (1.02 – 1.34), ***0.02*** |
| Sex (Female) | 413 | 206 (87%) | 141 (80%) | 1.75 (1.03 – 2.98), ***0.04*** |
| Disease indication [i.e. RA as Reference] | 387 | 44 (52%) | 41 (48%) | 0.76 (0.47 – 1.23), *0.26* |
| Concomitant DMARDs^1^, including HCQ | 387 | 189 (86%) | 123 (74%) | 2.18 (1.31-3.64), ***0.003*** |
| Concomitant oral prednisolone^2^ | 85 | 33 (75%) | 30 (73.2%) | 1.10 (0.42 – 2.90), *0.85* |
| Oral prednisolone dose (mg/day)^2^ | 85 | 13.7 (12.6) | 13.6 (11.6) | 1.00 (0.97 – 1.04), *0.96* |
| IgM (g/L) | 397 | 1.4 (0.7) | 1.5 (1.1) | 0.84 (0.67 – 1.05), *0.13* |
| IgA (g/L) | 398 | 3.0 (1.3) | 3.4 (1.6) | 0.82 (0.71 – 0.95), ***0.01*** |
| IgG (g/L) | 398 | 12.1 (4.1) | 13.6 (5.2) | 0.93 (0.89 – 0.98), ***0.003*** |
| Total B-cell counts  (x 10^9^/L)^3^ | 395 | 124.3 (125) | 143.4 (145) | 1.00 (1.00 – 1.00), *0.16* |
| Naïve B-cell counts  (x 10^9^/L)^3^ | 393 | 93.3 (96) | 106.2 (123) | 1.00 (1.00 – 1.01), *0.25* |
| Memory B-cell counts (x 10^9^/L)^3^ | 393 | 28.6 (54) | 32.1 (34) | 1.00 (0.99 – 1.00), *0.47* |
| Plasmablast counts  (x 10^9^/L)^3^ | 395 | 2.3 (3) | 5.8 (9) | 0.84 (0.78 – 0.89), ***<0.001*** |

^1^ Concomitant disease modifying anti-rheumatic drugs (DMARDs)

^2^ Data available for systemic lupus erythematosus (SLE) Leeds cohort only

^3^ Count x 10^9^ cells/L for each subset multiplied by 1000 prior to analysis

**Supplementary Table 10. Effect of *FCGR* genotype and copy number on complete B-cell depletion following rituximab in the combined RA and SLE analyses**

| ***Gene ^1^*** | **Genotype/**  **Copy Number** | **Complete B-cell Depletion: N (%)** | **Incomplete B-cell Depletion: N (%)** | **Unadjusted Complete B-cell Depletion post-RTX:**  **OR (95% CI), *p-value*^2^** | **Adjusted Complete B-cell Depletion post-RTX^3^:**  **OR (95% CI), *p-value*^2^** |
| --- | --- | --- | --- | --- | --- |
| **Genotypic Analyses** | | | | | |
| *FCGR2A*  (Q27W) | Q  (Reference) | 173 (73.3) | 138 (78.0) | - | - |
|  | QW | 59 (25.0) | 34 (19.2) | 1.38 (0.86 – 2.23), *0.18* | 1.22 (0.71 – 2.11), *0.46* |
|  | W | 4 (1.7) | 5 (2.8) | 0.64 (0.17 – 2.42), *0.51* | 1.28 (0.27 – 5.99), *0.76* |
|  | Additive model | - | - | 1.16 (0.78 – 1.73), *0.47* | 1.19 (0.75 – 1.89), *0.45* |
| *FCGR2A*  (H131R) | R  (Reference) | 63 (26.7) | 47 (26.6) | - | - |
|  | RH | 114 (48.3) | 94 (53.1) | 0.90 (0.57 – 1.44), *0.67* | 0.74 (0.43 – 1.27), *0.27* |
|  | H | 59 (25.0) | 36 (20.3) | 1.22 (0.70 – 2.14), *0.48* | 1.29 (0.67 – 2.48), *0.44* |
|  | Additive model | - | - | 1.10 (0.83 – 1.45), *0.52* | 1.12 (0.81 – 1.54), *0.48* |
| *FCGR3A*^4^ (F158V) | F  (Reference) | 95 (40.3) | 86 (48.6) | - | - |
|  | FV | 114 (48.3) | 80 (45.2) | 1.29 (0.86 – 1.94), *0.22* | 1.13 (0.70 - 1.82), *0.61* |
|  | V | 27 (11.4) | 11 (6.2) | 2.22 (1.04 – 4.75), ***0.04*** | 3.03 (1.23 – 7.42), ***0.02*** |
|  | Additive model | - | - | 1.40 (1.03 – 1.91), ***0.03*** | 1.44 (1.01 – 2.06), ***0.04*** |
| *FCGR2C*^4^  (STP/ORF) | STP  (Reference) | 156 (70.0) | 126 (75.0) | - | - |
|  | STPORF | 59 (26.5) | 36 (21.4) | 1.32 (0.82 – 2.13), *0.25* | 1.28 (0.75 - 2.21), *0.36* |
|  | ORF | 8 (3.6) | 6 (3.6) | 1.08 (0.36 – 3.18), *0.89* | 1.51 (0.41 – 5.58), *0.54* |
|  | Additive model | - | - | 1.20 (0.82 – 1.75), *0.36* | 1.26 (0.81 – 1.96), *0.30* |
| *FCGR3B*^4^  (NA1/NA2 haplotype) | NA2  (Reference) | 98 (44.0) | 65 (40.4) | - | - |
|  | NA2NA1 | 98 (44.0) | 75 (46.6) | 0.87 (0.56 – 1.34), *0.52* | 1.04 (0.62 - 1.74), *0.88* |
|  | NA1 | 27 (12.1) | 21 (13.0) | 0.85 (0.44 – 1.63), *0.63* | 0.74 (0.36 – 1.55), *0.43* |
|  | Additive model | - | - | 0.91 (0.67 – 1.22), *0.52* | 0.90 (0.64 – 1.28), *0.57* |
| *FCGR2B*  (I123T) | I  (Reference) | 184 (78.3) | 137 (78.3) | - | - |
|  | IT | 48 (20.4) | 36 (20.6) | 0.99 (0.61 – 1.61), *0.98* | 0.87 (0.49 - 1.54), *0.63* |
|  | T | 3 (1.3) | 2 (1.1) | 1.12 (0.18 – 6.78), *0.90* | 0.43 (0.06 – 3.21), *0.41* |
|  | Additive model | - | - | 1.01 (0.65 – 1.56), *0.98* | 0.82 (0.49 – 1.36), *0.44* |
| **Copy Number Analyses** | | | | | |
| *FCGR3A* (F158V) | 2 copies (reference) | 208 (88.1) | 165 (93.2) | - | - |
|  | <2 copies | 5 (2.1) | 1 (0.6) | 3.97 (0.46 – 34.28), *0.21* | 3.12 (0.33 – 29.57), *0.32* |
|  | >2 copies | 23 (9.8) | 11 (6.2) | 1.66 (0.79 – 3.50), *0.18* | 2.35 (0.99 – 5.57), ***0.05*** |
|  | Per copy of V allele | 141 (59.7) | 91 (51.4) | 1.41 (1.05 – 1.90), ***0.02*** | 1.49 (1.06 – 2.08), ***0.02*** |
| *FCGR2C* (STP/ORF) | 2 copies (reference) | 172 (77.1) | 135 (80.4) | - | - |
|  | <2 copies | 22 (9.9) | 12 (7.1) | 1.44 (0.69 – 3.01), *0.33* | 1.23 (0.52 – 2.88), *0.64* |
|  | >2 copies | 29 (13.0) | 21 (12.5) | 1.08 (0.59 – 1.99), *0.79* | 1.28 (0.65 – 2.54), *0.48* |
|  | Per copy of ORF allele | 67 (30.0) | 42 (25.0) | 1.23 (0.87 – 1.75), *0.24* | 1.35 (0.90 – 2.02), *0.15* |
| *FCGR3B* (NA1/NA2 haplotype) | 2 copies (reference) | 191 (80.9) | 146 (82.5) | - | - |
|  | <2 copies | 22 (9.3) | 14 (7.9) | 1.20 (0.59 – 2.43), *0.61* | 1.13 (0.51 – 2.54), *0.76* |
|  | >2 copies | 23 (9.8) | 17 (9.6) | 1.03 (0.53 – 2.01), *0.92* | 1.14 (0.54 – 2.40), *0.73* |
|  | Per copy of NA1 allele | 131 (57.2) | 104 (61.5) | 0.85 (0.64 – 1.14), *0.28* | 0.91 (0.65 – 1.27), *0.56* |

^1^Genes presented in chromosomal order on 1q23 centromere to telomere.

^2^Odds ratio (OR), 95% confidence intervals (CI) and p-value for the effect of the indicated genotype or copy number on complete B-cell depletion at 2 weeks, compared with reference genotype. All tests were performed using logistic regression.

^3^Analyses adjusted for age, concomitant disease-modifying anti-rheumatic drug, including hydroxychloroquine, and baseline plasmablast count.

*^4^FCGR3A, FCGR2C* and *FCGR3B* are subject to copy number variation, analyses were performed according to biallelic genotype whereby the effect of heterozygosity and homozygosity for the rare allele were compared with homozygosity for the common allele.

N: number

**Appendix I**

**MATURA Consortium**

**Work Stream 1:** Prof Costantino Pitzalis (Queen Mary University of London), Prof Peter Taylor (University of Oxford), Prof Ernest Choy (Cardiff University), Prof Iain McInnes (University of Glasgow), Prof Michael Barnes (Queen Mary University of London), Prof John Isaacs (Newcastle University), Prof Christopher Buckley (University of Birmingham), Prof Michael Ehrenstein (University College London), Prof Peter Sasieni (Queen Mary University of London), Dr Andrew Filer (University of Birmingham).

**Work Stream 2:** Prof Anne Barton (University of Manchester), Prof Ann Morgan (University of Leeds), Prof Gerry Wilson (University College Dublin), Prof Paul McKeigue (University of Edinburgh), Prof Heather Cordell (Newcastle University), Prof Jenny Barrett (University of Leeds), Prof Andrew Cope (Kings College London), Prof Adam Young (University of Hertfordshire), Prof Karim Raza (University of Birmingham), Prof Katherine Payne (University of Manchester), Prof Jane Worthington (University of Manchester), Prof Deborah Symmons (University of Manchester), Prof Kimme Hyrich (University of Manchester). Industry: Martin Hodge (Pfizer), Anthony Rowe (Janssen), Jianmei Wang (Roche/Genentech), Michelle Mao (BGI), Patricia McLoughlin (Qiagen), Carolyn Cuff (AbbVie), David Close (MedImmune).

**Leeds Biologics Service (University of Leeds and Leeds Teaching Hospitals NHS Trust)**

**Management Team:** Paul Emery, Maya Buch, Elizabeth MA Hensor

**Consultant and Senior Scientific Staff:** Maya Buch, Paul Emery, Ann Morgan, Frederique Ponchel, Shouvik Dass, Edward Vital, Sarah Bingham

**Specialist Registrars and Clinical Fellows:** Edith Villeneuve, Sudipto Das, Jacqueline Nam, Sarah Horton, Sarah Mackie, Benazir Saleem, Rebecca Thomas, Lesley-Anne Bissell, Chadi Rakieh, Zoe Ash, Sarah Twigg, Laura Coates, Fahad Fazal, Laura Hunt, Esme Ferguson, Kavitha Nadesalingam, Sara Else, Gui Tran, Ahmed Zayat, Giuseppina Abignano, Md Yuzaiful Md Yusof, Radhika Raghunath, Hannah Mathieson, Chitra Salem-Ramakumaran, Hanna Gul, Mahwish Mahmood, Leticia Garcia-Montoya, Jean-Baptiste Candelier, Thibault Rabin, Gisela Eugenio, Joana Fonseca Ferreira.

**Biologics Nursing Staff:** Pauline Fitzgerald, Matthew Robinson, Jason Ward, Beverly Wells, David Pickles, Oliver Wordsworth, Christine Thomas, Alison McManus, Lynda Bailey, Linda Gray, Katherine Russell, Jayne Davies

**Laboratory and Support Staff:** Diane Corscadden, Karen Henshaw, Katie Mbara, Stephen Martin, James Robinson, Dawn Wild, Agata Burska, Sarah Fahey, Jill Halsted-Rastrick, Ged Connoly-Thompson, Jonathan Thompson, Ian Weatherill, Andrea Paterson

**IACON Radiology team:** Richard Wakefield, Laura Horton, Alwyn Jackson, Richard Hodgson

**Appendix II**

**Contributors to the MASTERPLANS Consortium**

The University of Manchester: Prof Katherine Payne; Dr Mark Lunt; Prof Niels Peek; Dr Nophar Geifman; Dr Sean Gavan; Dr Gillian Armitt; Dr Patrick Doherty; Dr Jennifer Prattley; Dr Narges Azadbakht; Angela Papazian; Dr Helen Le Sueur; Carmen Farrelly; Clare Richardson; Zunnaira Shabbir; Lauren Hewitt; Dr Emily Sutton; Alison Fountain, Ilina Serafimova.

University of Bath: Prof Neil McHugh.

University of Birmingham: Prof Caroline Gordon; Prof Stephen Young.

University of Cambridge: Prof David Jayne; Prof Vern Farewell; Dr Li Su.

Imperial College London: Prof Matthew Pickering; Prof Elizabeth Lightstone; Dr Alyssa Gilmore; Prof Marina Botto.

King's College London: Prof Timothy Vyse; Dr David Lester Morris; Prof David D’Cruz.

University of Liverpool: Prof Michael Beresford; Prof Christian Hedrich; Dr Angela Midgley; Dr Jenna Gritzfeld.

University College London: Prof Michael Ehrenstein; Prof David Isenberg; Mariea Parvaz.

MASTERPLANS Patient and Public Involvement Group: Jane Dunnage; Jane Batchelor; Elaine Holland; Pauline Upsal
