# Supplementary material for: Comprehensive genetic and functional analyses of Fc gamma receptors influence on response to rituximab therapy for autoimmunity
Source: eBioMedicine. 2022 Nov 11;86:104343. doi: 10.1016/j.ebiom.2022.104343 (PMC9663864; doi:10.1016/j.ebiom.2022.104343)
Supplement: Supplementary File S1 [file mmc3.docx]

**Captions for Supplementary Figures**

**Supplementary Figure 1. Sequencing-based *FCGR2C* QSV copy number assay.** *FCGR2C QSV* exploits a unique paralogous sequence variant (arrowed) discriminating *FCGR2A*, *B* and *C* (hg19 Chr1:161,481,793; 161,645,283: 161,563,452 respectively). This variant is also known as NCBI dbSNP rs569867770. **(a)** Simultaneous PCR amplification of all three genes using common primers enabled proportional quantification of copy number variable *FCGR2C* with reference to the copy number invariable *FCGR2A* and *FCGR2B* from Sanger sequencing electropherograms using QSVanalyser. **(b)** Copy number calls based on the observed clustering were used to interpret *FCGR2C* STP/ORF quantitative genotypes from multiplexed ligation-dependent probe amplification panels P110 and P111 (version B2).

**Supplementary Figure 2. Natural Killer cell flow cytometric analysis. (a)** Peripheral blood NK-cells were identified on the basis of lymphocyte forward scatter/side scatter characteristics and gating for lack of CD3 expression and positive expression of CD56 (CD3^neg^/CD56^pos^). **(b)** NK-cell subsets were distinguished by separate gates created around CD56^dim^/CD16^++^ and CD56^bright^/CD16^neg/low^ NK-cells.

**Supplementary Figure 3. NK-cell-mediated antibody dependent cellular cytotoxicity assay.** The figure shows cell surface CD107a, gated on CD3-CD56+ natural killer (NK)-cells within peripheral blood mononuclear cells when co-cultured with Raji target cells using E:T ratio of 1:1 and in the absence of rituximab (left hand panel) and the presence of rituximab (right hand panel). The percentage values indicate the proportion of CD107+NK-cells for each *FCGR3A* genotype in systemic lupus erythematosus (SLE) and healthy control. Degranulation activity was measured by the ratio of NK-cell degranulation with and without rituximab.

**Supplementary Figure 4: Flow chart of patients with RA and SLE included into the study.** 2C-DAS28CRP: two-component disease activity score in 28-joints, BILAG-BR: British Isles Lupus Assessment Group Biologics Registry, BRAGGSS: Biologics in Rheumatoid Arthritis Genetics and Genomics Study Syndicate, BSRBR-RA: British Society for Rheumatology Biologics Register for Rheumatoid Arthritis.

**Supplementary Figure 5. FcγRIIIa expression on NK-cell subsets in healthy controls, RA and SLE.** Natural killer (NK)-cell (CD3-CD56+CD16+) FcγRIIIa (CD16 clone 3G8) geometric mean fluorescence intensity (MFI) using flow cytometry for healthy controls (HC), early (symptom onset <1 year and treatment naïve) and established (>2 years) rheumatoid arthritis (RA). **(a)** Percentage of NK-cells in the CD56^bright^ subset for HC (n=28), early RA (n=43) and established RA (n=20). **(b)** FcγRIIIa expression on CD56^dim^ NK-cells for HC (n=43), early RA (n=27) and established RA (n=20). **(c)** FcγRIIIa expression on CD56^bright^ NK-cells for HC (n=42), early RA (n=28) and established RA (n=20). **(d)** NK-cell FcγRIIIa expression in HC (n=9) and systemic lupus erythematosus (SLE) (n=10) patients prior to rituximab treatment. FcRγIIIa expression on NK-cells in different *FCGR3A*-F158V genotypes in HC (n=30) **(e)**, RA (n=46) **(f)** and SLE (n=13) patients **(g)**, with two copies of *FCGR3A*. **(h)** Frequency of NK cells (CD3-CD56+) between RA (n=18) and SLE (n=17) patients. All p-values calculated using non-parametric Mann-Whitney (bars with dropdowns) and Kruskal-Wallis H test (bars without dropdowns). Data are summarised using median and the error bars denote interquartile range.
