# Supplementary material for: Comprehensive genetic and functional analyses of Fc gamma receptors influence on response to rituximab therapy for autoimmunity
Source: eBioMedicine. 2022 Nov 11;86:104343. doi: 10.1016/j.ebiom.2022.104343 (PMC9663864; doi:10.1016/j.ebiom.2022.104343)
Supplement: Supplementary File S2 [file mmc4.docx]

| **MASTERPLANS Consortia** | |
| --- | --- |
| Mark | Lunt |
| Niels | Peek |
| Nophar | Geifman |
| Sean | Gavan |
| Gillian | Armitt |
| Patrick | Doherty |
| Jennifer | Prattley |
| Narges | Azadbakht |
| Angela | Papazian |
| Helen | Le Sueur |
| Carmen | Farrelly |
| Claire | Richardson |
| Zunnaira | Shabbir |
| Lauren | Hewitt |
| Emily | Sutton |
| Alison | Fountain |
| Ilina | Serafimova |
| Neil | McHugh |
| Caroline | Gordon |
| Stephen | Young |
| David | Jayne |
| Vern | Farewell |
| Li | Su |
| Matthew | Pickering |
| Elizabeth | Lightstone |
| Alyssa | Gilmore |
| Marina | Botto |
| David | D’Cruz |
| Michael | Beresford |
| Christian | Hedrich |
| Angela | Midgley |
| Jenna | Gritzfeld |
| David | Isenberg |
| Mariea | Parvaz |
| Jane | Dunnage |
| Jane | Batchelor |
| Elaine | Holland |
| Pauline | Upsal |

Please note that First names will be abbreviated in PubMed.

###
