# Supplementary material for: Comprehensive genetic and functional analyses of Fc gamma receptors influence on response to rituximab therapy for autoimmunity
Source: eBioMedicine. 2022 Nov 11;86:104343. doi: 10.1016/j.ebiom.2022.104343 (PMC9663864; doi:10.1016/j.ebiom.2022.104343)
Supplement: Supplementary File S3 [file mmc5.docx]

| **MATURA Consortia** | |
| --- | --- |
| Constantino | Pitzalis |
| Peter | Taylor |
| Ernest | Choy |
| Iain | McInnes |
| Michael | Barnes |
| Christopher | Buckley |
| Michael | Ehrenstein |
| Peter | Sasieni |
| Andrew | Filer |
| Gerry | Wilson |
| Paul | McKeigue |
| Andrew | Cope |
| Adam | Young |
| Karim | Raza |
| Katherine | Payne |
| Jane | Worthington |
| Deborah | Symmons |
| Kimme | Hyrich |
| Martin | Hodge |
| Anthony | Rowe |
| Jianmei | Wang |
| Michelle | Mao |
| Patricia | McLoughlin |
| Carolyn | Cuff |
| David | Close |
| Elizabeth MA | Hensor |
| Frederique | Ponchel |
| Shouvik | Dass |
| Sarah | Bingham |
| Edith | Villeneuve |
| Sudipto | Das |
| Jacqueline | Nam |
| Sarah | Horton |
| Sarah | Mackie |
| Benazir | Saleem |
| Rebecca | Thomas |
| Lesley-Anne | Bissell |
| Chadi | Rakieh |
| Zoe | Ash |
| Sarah | Twigg |
| Laura | Coates |
| Fahad | Fazal |
| Laura | Hunt |
| Esme | Ferguson |
| Kavitha | Nadesalingam |
| Sara | Else |
| Gui | Tran |
| Ahmed | Zayat |
| Giuseppina | Abignano |
| Radhika | Raghunath |
| Hannah | Mathieson |
| Chitra | Salem-Ramakumaran |
| Hanna | Gul |
| Mahwish | Mahmood |
| Leticia | Garcia-Montoya |
| Jean-Baptiste | Candelier |
| Thibault | Rabin |
| Gisela | Eugenio |
| Joana Fonseca | Ferreira |
| Pauline | Fitzgerald |
| Matthew | Robinson |
| Jason | Ward |
| Beverly | Wells |
| David | Pickles |
| Oliver | Wordsworth |
| Christine | Thomas |
| Alison | McManus |
| Lynda | Bailey |
| Linda | Gray |
| Katherine | Russell |
| Jayne | Davies |
| Diane | Corscadden |
| Karen | Henshaw |
| Katie | Mbara |
| Stephen | Martin |
| Agata | Burska |
| Sarah | Fahey |
| Jill | Halstead-Rastrick |
| Ged | Connoly-Thompson |
| Jonathan | Thompson |
| Ian | Weatherill |
| Andrea | Patterson |
| Richard | Wakefield |
| Laura | Horton |
| Alwyn | Jackson |
| Richard | Hodgson |
